# Spinal lumbar dI2 interneurons contribute to stability of bipedal stepping

**DOI:** 10.7554/eLife.62001

**Published:** 2021-08-16

**Authors:** Baruch Haimson, Yoav Hadas, Nimrod Bernat, Artur Kania, Monica A Daley, Yuval Cinnamon, Aharon Lev-Tov, Avihu Klar

**Affiliations:** 1 Department of Medical Neurobiology, IMRIC, Hebrew University – Hadassah Medical School Jerusalem Israel; 2 Institut de recherches cliniques de Montréal (IRCM) Montréal Canada; 3 Ecology and Evolutionary Biology, University of California, Irvine Irvine United States; 4 Institute of Animal Science Poultry and Aquaculture Sci. Dept. Agricultural Research Organization, The Volcani Center Rishon LeZion Israel; CNRS Université de Bordeaux France; Emory University United States

**Keywords:** spinal cord, interneurons, locomotion, cerebellum, spinocerebellar tract, neural circuits, Chicken

## Abstract

Peripheral and intraspinal feedback is required to shape and update the output of spinal networks that execute motor behavior. We report that lumbar dI2 spinal interneurons in chicks receive synaptic input from afferents and premotor neurons. These interneurons innervate contralateral premotor networks in the lumbar and brachial spinal cord, and their ascending projections innervate the cerebellum. These findings suggest that dI2 neurons function as interneurons in local lumbar circuits, are involved in lumbo-brachial coupling, and that part of them deliver peripheral and intraspinal feedback to the cerebellum. Silencing of dI2 neurons leads to destabilized stepping in posthatching day 8 hatchlings, with occasional collapses, variable step profiles, and a wide-base walking gait, suggesting that dI2 neurons may contribute to the stabilization of the bipedal gait.

## Introduction

The spinal cord integrates and relays the somatosensory inputs required for further execution of complex motor behaviors. Interneurons (INs) that differentiate at the ventral progenitor domain, V3-V0, are involved in the control of rhythmic motor activity, alternating between the left and right limbs, as well as between the flexor and extensor muscles (Lai et al., 2016; Osseward and Pfaff, 2019; Alaynick et al., 2011). Some of the dorsally born INs, dI1, dI3, and dI6, migrate ventrally and are also assembled within circuitries that control motor activity (Yuengert et al., 2015; Bui et al., 2013; Andersson et al., 2012), while other dorsal progenitor neurons, dI4 and dI5, give rise to INs that mediate somatosensation (Lai et al., 2016).

dI2 neurons originate in the dorsal spinal cord. The progenitor pdI2 cells, topographically positioned between the adjacent dorsally located dI1 and ventrally located dI3 neurons, express Ngn1, Ngn2, Olig3, and Pax3 transcription factors (TFs). Early postmitotic dI2 neurons undergo ventral migration and are defined by the combinatorial expression of Foxd3^+^/Lhx1^+^/Pou4f1^+^ TFs (Alaynick et al., 2011; Morikawa et al., 2009; Francius et al., 2013). Importantly, none of these TFs is specific to dI2 neurons; rather, their combinatorial expression defines dI2. The lack of dI2-specific cell fate markers and the dynamic expression of the above TFs in other INs causes ambiguity regarding the molecular profile and outcome of late postmitotic dI2 neurons. Using intersectional genetics, we have shown previously that dI2 neurons are commissurally projecting neurons (Avraham et al., 2009). The lack of dI2-specific TFs impeded their genetic targeting. Hence, little is known about the wiring and physiological function of dI2 neurons.

The maintenance of stability and the coordination, precision, and timing of movements are regulated and modulated by the cerebellum. Anatomical and electrophysiological studies of cats and rodents revealed two major pathways ascending from neurons in the lumbar spinal cord to the cerebellum: the dorsal spinocerebellar tract (DSCT) and ventral spinocerebellar tract (VSCT). DSCT neurons are considered to relay mainly proprioceptive information, while VSCT neurons are thought to relay internal spinal network information to the cerebellum along with proprioceptive data (Jankowska and Hammar, 2013; Spanne and Jörntell, 2013; Stecina et al., 2013; Jiang et al., 2015). While subpopulations of DSCT neurons are genetically accessible (Hantman and Jessell, 2010), the genetic inaccessibility of VSCT neurons hinders efforts to reveal their actual contribution to the regulatory functions of the cerebellum in locomotion and other motor behaviors.

In the present study, we investigated the possible functions of dI2 neurons in chick motor behavior. Employing intersectional genetics in the chick spinal cord, we targeted dI2 neurons and found evidence implicating them in the control of stability during locomotion. There are several advantages to performing these studies in chicks. The patterning of neurons within the spinal cord (Jessell, 2000) and the spinocerebellar tracts (Furue et al., 2010; Furue et al., 2011; Uehara et al., 2012) is conserved between mammals and birds. In addition, chicks use bipedal locomotion (evolved in humans and birds) that can be examined soon after hatching. To decode the circuitry and function of spinal INs, we developed a unique circuit-deciphering toolbox that enables neuron-specific targeting and tracing of circuits in the chick embryo (Hadas et al., 2014), and we utilized kinematic analysis of overground bipedal stepping by the hatched chicks following silencing of dI2 neurons.

Our studies revealed that lumbar dl2 neurons receive synaptic inputs from inhibitory and excitatory premotor neurons (pre-MNs) and relay output to the cerebellar granular layer, pre-MNs in the contralateral spinal cord and the contralateral dI2 cells. In a kinematic analysis of overground stepping by posthatching day (P) 8 hatchlings after inhibition of dI2 neuronal activity by expression of the tetanus toxin light chain gene, the genetically manipulated hatchlings showed an unstable gait, demonstrating that dI2 neurons play a role in shaping and stabilizing the bipedal gait.

## Results

To define potential VSCT neurons within spinal INs, we defined the following criteria: (1) soma location in accordance with precerebellar neurons at the lumbar level, which were previously revealed by retrograde labeling experiments of the chick cerebellar lobes (Furue et al., 2010; Furue et al., 2011; Uehara et al., 2012), (2) commissural neurons, (3) excitatory neurons, and (4) non-pre-MNs (Lai et al., 2016; Osseward and Pfaff, 2019; Alaynick et al., 2011). Based on these criteria, dI1c and dI2 neurons are likely candidates (Bermingham et al., 2001; Yuengert et al., 2015; Figure 1—figure supplement 1). This is further supported by Sakai et al., 2012, who demonstrated that in the embryonic day (E) 12 chick, the dI1 and dI2 axons project to the hindbrain and toward the cerebellum. In the current study, we focused on deciphering the circuitry and function of dI2 neurons and their possible association with VSCT.

### dI2 INs are mainly excitatory neurons with commissural axonal projections

**Figure 1.**
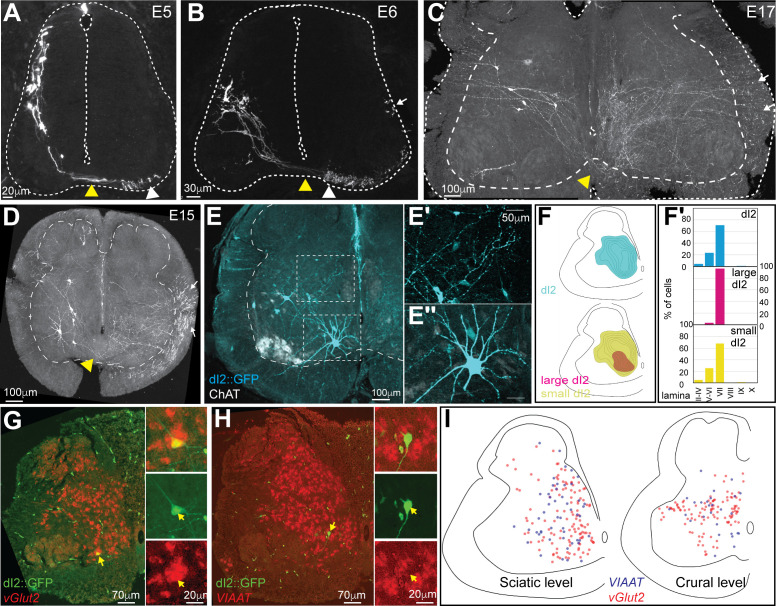
Characterization and classification of dI2 neurons during embryonic development. dI2 interneurons (INs) were labeled as cells that expressed both the Foxd3 and Ngn1 enhancers (Avraham et al., 2009; see Figure 1—figure supplement 1A). (**A–D**) dI2 axonal projection during development. At embryonic day (E) 5 (**A**), postmitotic dI2 neurons assume a dorsolateral position and start to migrate ventrally. At E6 (**B**), dI2 neurons occupy the midlateral domain. At E15-17, dI2 neurons are located at medial lamina VII at the lumbar level (LS3) (**C**) and thoracic level (T1) (**D**). dI2 axons cross the floor plate (yellow arrowheads), turn longitudinally at the ventral funiculus (white arrowheads) and eventually elongate at the lateral funiculus (white arrows). (**E**) Cross-section of an E17 embryo at the lumbar spinal cord (crural plexus level, LS2). Small-diameter dI2 neurons residing in lamina VII (**E′**) and ventromedial large-diameter dI2 neurons in lamina VIII (**E″**). (**F**) Density plots and laminar distribution (**F′**) of dI2 somata at the sciatic plexus level (cyan, N = 374 cells); large-diameter (magenta) and small-diameter dI2 (yellow) INs (N = 33 and N = 344 cells, respectively, from two embryos). (**G, H**) Neurotransmitter phenotype of dI2 neurons. dI2 neurons expressing GFP were subjected to in situ hybridization using the *Vglut2* probe (**G**) or the *VIAAT* probe (**H**). (**I**) Distribution of excitatory (vGlut2, red) and inhibitory (VIAAT, blue) dI2 neurons at the sciatic and crural levels at E17 (N = 172 and 136 neurons, respectively, from two embryos). See Figure 1—source data 1 and 2. Figure 1—source data 1.Localization of dI2 neurons at the sciatic level.The X/Y coordinates of dI2, large-diameter dI2, and small-diameter dI2 neurons (Figure 1F). Figure 1—source data 2.Localization of excitatory and inhibitory dI2 neurons.The X/Y coordinates of dI2 neurons expressing either vGlut2 or VIATT (Figure 1I).

To label dI2 neurons, axons, and terminals in the chick spinal cord, we used intersection between enhancers of two TFs expressed by dI2 – Ngn1 and Foxd3 – via the expression of two recombinases (Cre and FLPo) and double conditional reporters (Figure 1—figure supplement 1A). We have shown previously that the combination of these enhancers reliably labels dI2 neurons (Avraham et al., 2009; Hadas et al., 2014). The recombinases and the double conditional reporter plasmids were delivered via spatially restricted electroporation to the lumbar spinal cord at HH18. At E5, early postmitotic dI2 neurons migrate ventrally from the dorsolateral to the midlateral spinal cord (Figure 1A). As they migrate ventrally, at E6, dI2 neurons assume a midlateral position along the dorsoventral axis (Figure 1B). Subsequently, dI2 neurons migrate medially, and at E17, comparable to postnatal day 4 (P4) of mice, most of them (70.7 and 71.5% at the sciatic and the crural levels, respectively) occupy lamina VII (Figure 1C and F). At all rostrocaudal levels and embryonic stages, dI2 axons cross the floor plate (Figure 1A–D). After crossing, dI2 axons extend rostrally for a few segments in the ventral funiculus (VF) and subsequently turn into the lateral funiculus (LF) (Avraham et al., 2009; Figure 1C and D). Collaterals originating from the crossed VF and LF tracts invade the contralateral spinal cord (Figure 1C and D; white arrows).

**Figure 1—figure supplement 1.**
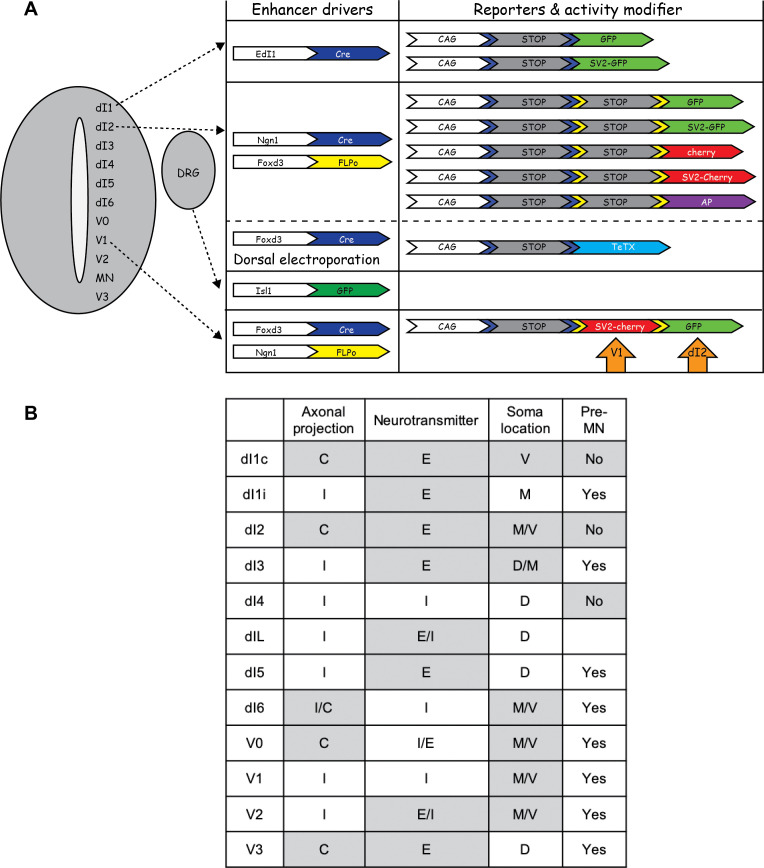
Targeting, reporters, and activity modifiers used in the study. (**A**) The different DNA constructs used in this study. For targeted expression in dI1 cells, the EdI1 enhancer was used (Avraham et al., 2009). To target dI2 cells, the intersection of the *Ngn1* and *Foxd3* enhancers was attained (Avraham et al., 2009; Hadas et al., 2014). The Isl1 enhancer was used for targeted expression in DRG neurons (Uemura et al., 2005). (**B**) A table summarizing the axonal projections, neurotransmitter identity, soma location, and existence of synaptic connections between premotor neurons (pre-MNs) and spinal interneuron (IN) populations. Based on previous studies (Jessell, 2000; Alaynick et al., 2011; Lai et al., 2016).

**Figure 1—figure supplement 2.**
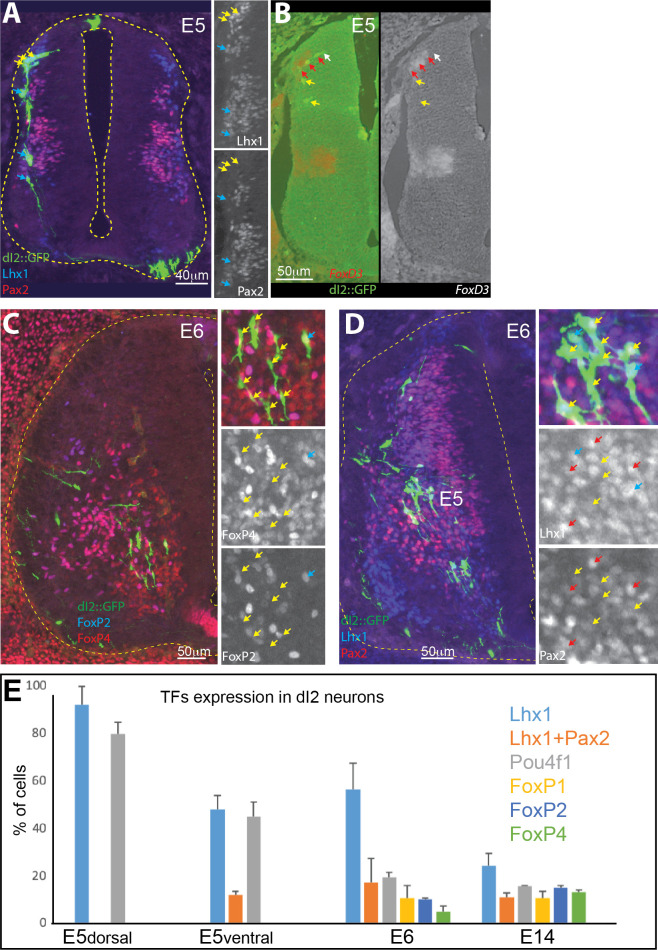
Differential expression of transcription factor (TF) in dI2 neurons. (**A**) Premigratory dI2 (yellow) and early ventral migrating dI2 (cyan) neurons are Lhx1^+^Pax2^-^ (cyan arrows). Cross-section of an embryonic day (E) 5 chick neural tube, expressing GFP in dI2 neurons and immunostained for Lhx1 and Pax2 (insets at the right side). (**B**) Postmitotic, premigratory dI2 neurons express *FoxD3* (red arrows) and downregulate its expression upon ventral migration (yellow arrows). The white arrow points to early postmitotic dI2 neurons that have not yet begun to express *FoxD3*. Cross-section of a chick neural tube at E5, double labeled for dI2 (dI2::GFP) and processed for in situ hybridization with a *FoxD3* probe. (**C**) Genetic heterogeneity in the dI2 neuron population. Only a small subset of dI2 neurons express both the *FoxP2* and *FoxP4* genes. The cyan arrow indicates a *FoxP2*^+^/*FoxP4*^+^ dI2::GFP neuron. The yellow arrows point to FoxP2^-^/FoxP4^-^ neurons. Cross-section of an E6 chick neural tube expressing GFP in dI2 interneurons (INs) and immunostained for FoxP2 and FoxP4. (**D**) A fraction of dI2 neurons express Pax2, which indicates an inhibitory phenotype. Cross-section of an E6 chick neural tube expressing GFP in dI2 INs and immunostained for Lhx1 and Pax2. The red arrows point to dI2::GFP/Lhx1^+^/Pax2^+^, the cyan arrows to Lhx1^+^/Pax2^-^, and the yellow arrows to Lhx1^-^/Pax2^-^ dI2 neurons. (**E**) A graph indicating the fraction of dI2 neurons expressing TFs during development (based on data from three E5, two E6, and two E14 embryos). Figure 1—figure supplement 2—source data 1.Pattern of expression of transcription factors (TFs) in dI2 neurons.Expression of TFs in dI2 neurons (Figure 1—figure supplement 2E).

**Figure 1—figure supplement 3.**
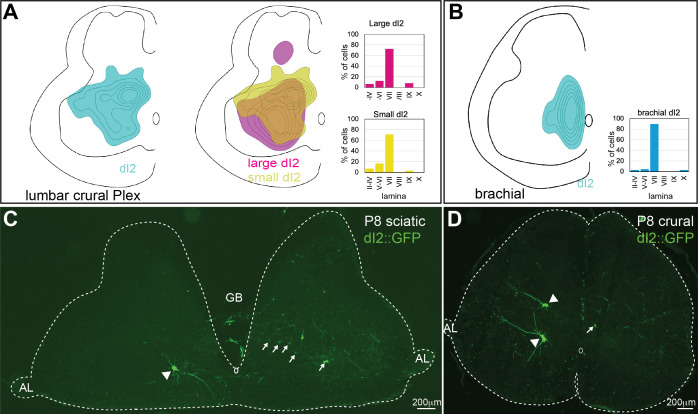
Distribution of dI2 neurons at the embryonic and posthatching spinal cord. (**A**) Density plot and laminar distribution of dI2 somata in crural plexus segments (N = 551 cells) (left). Density plot of dI2_large_ (magenta) and dI2_small_ (yellow) neurons in the crural plexus segments (N = 48 and N = 502 cells, respectively; N = 2 embryos) (right). (**B**) Density plot and laminar distribution of dI2 soma in the brachial segments (N = 66 cells, N = 2 embryos). (**C, D**) dI2 neurons in P8 chick hatchlings. dI2 neurons were labeled by the FoxD3 enhancer and bilateral dorsal electroporation (Figure 1—figure supplement 1A). Large-diameter dI2 neurons are indicated by arrowheads and some of the small-diameter dI2 by arrows. Figure 1—figure supplement 3—source data 1.Localization of dI2 neurons at the crural and brachial levels.The X/Y coordinates of large diameter dI2 and small diameter dI2 neurons in the crural and brachial spinal levels (Figure 1—figure supplement 3A and B).

A recent study suggested that dI2 neurons at early stages of development in mice (E9.5–E13.5, comparable to chick E4–8) can be divided into several subclasses based on their genetic signature and degree of maturation (Delile et al., 2019). To assess the diversity of dI2 neurons in the chick, the expression of dI2 TFs in dI2::GFP cells was analyzed at E5 before and during ventral migration, at E6 and E14. The early postmitotic dI2::GFP cells at E5 were a homogenous population defined by Foxd3^+^/Lhx1^+^/Pou4f1^+^/Pax2^-^ (Figure 1—figure supplement 2A, B, E). dI2 neurons that underwent ventral migration at E5, as well as at E6 and E14, express variable combinations of Lhx1, Pou4f1, and FoxP1/2/4 (Figure 1—figure supplement 2C–E). At E14, approximately 50% of dI2::GFP cells did not express any of the tested TFs (Figure 1—figure supplement 2E), suggesting that the early expression of TFs is required for cell fate acquisition, axon guidance, and target recognition, while their expression is not required after the establishment of the circuitry, as shown for other spinal INs (Bikoff et al., 2016). Interestingly, approximately 12% of ventrally migrating dI2 neurons (from E5 to E14) expressed Pax2 (Figure 1—figure supplement 2D and E). Pax2 is associated with a GABAergic inhibitory phenotype (Cheng et al., 2004), suggesting that a subpopulation of dI2 are inhibitory neurons. The distribution of excitatory and inhibitory dI2 neurons is also apparent at E17. In situ hybridization on cross-sections of the E17 dI2::GFP-labeled lumbar spinal cord using the vGlut2 probe revealed that 73% were vGlut2^+^, while the VIAAT probe, which labels GABAergic and glycinergic inhibitory neurons, measured 27% VIAAT^+^ dI2 neurons (Figure 1G–I; N = 308 neurons from two embryos). Similar percentages of Gad2- and Slc6a5-dI2-expressing cells were also found in mice (Delile et al., 2019). At E13–17 at the caudal lumbar level and at the level of the sciatic plexus, most dI2 neurons are located in the medial aspect of lamina VII. Approximately 91% of dI2 neurons are small-diameter neurons located in the lateral dorsal aspect of lamina VII, and 9% are large-diameter neurons. At the lumbar sciatic plexus level, large-diameter dI2 neurons are located mostly at the ventral aspect of lamina VII (Figure 1F) and at the level of the crural plexus in the ventral and dorsal aspect of lamina VII (Figure 1—figure supplement 3A). The location of the lumbar dI2 neurons, mainly lamina VII, is also apparent in posthatching chicks (P8, Figure 1—figure supplement 3C and D). Importantly, large-diameter dI2 neurons were apparent only at the lumbar level (Figure 1F, Figure 1—figure supplement 3A). The division of large- and small-diameter lumbar dI2 neurons was not reflected in the expression of the tested TFs or in a specific neurotransmitter phenotype; the inhibitory/excitatory ratios were 0.297 ± 0.13 and 0.343 ± 0.02, for large- and small-diameter dI2 neurons, respectively. Hence, dI2 neurons consist of several subpopulations, as has been shown in other spinal INs (Bikoff et al., 2016; Delile et al., 2019; Sweeney et al., 2018).

### Subpopulation of dI2 neurons project to the cerebellum

**Figure 2.**
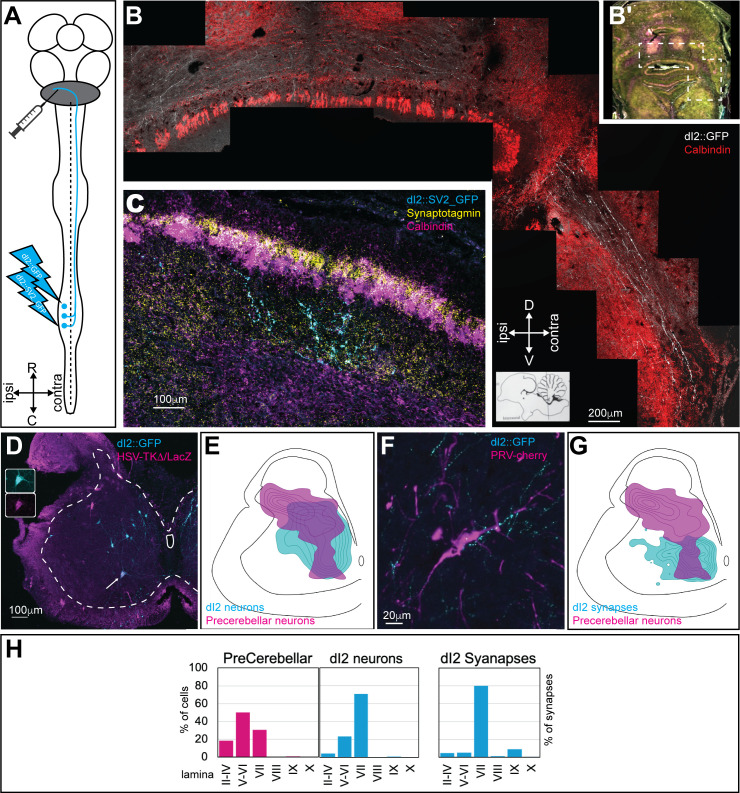
dI2 neurons project to the cerebellum. (**A**) Experimental setup for labeling dI2 neurons that project to the cerebellum. dI2 neurons were genetically targeted at HH18, and precerebellar neurons were labeled using intracerebellar injection of replication-defective HSV-LacZ or PRV-Cherry at embryonic day (E) 15. The abbreviations in the coordinates: R: rostral; C: caudal. (**B**) A cross-section of E17 brainstem and cerebellum. The dashed polygon in (**B′**) is magnified in (**B**). dI2 axons reach the cerebellum, enter into it via the superior cerebellar peduncle, and cross the cerebellar midline. Calbindin (Purkinje neurons, magenta [**B′**] or red [**B**]). Abbreviations in the coordinates: D: dorsal; V: ventral. (**C**) A cross-section of E17 cerebellar cortex. Lumbar-originating dI2 synapses (cyan) in the granular layer of the anterior cerebellar cortex. Calbindin (Purkinje neurons, magenta), synaptotagmin (yellow). (**D**) A cross-section of an E15 embryo at the lumbar spinal cord level (sciatic plexus level). Precerebellar neurons were infected and labeled with HSV-LacZ (magenta), and dI2 neurons expressed GFP (cyan). A large-diameter dI2 neuron coexpressing LacZ and GFP is indicated by an arrow (magnification of the two channels in the insets). (**E**) Density plots of dI2 and precerebellar neurons (density values 10–90%) in the sciatic plexus segments (N = 374 and N = 289 cells, respectively). (**F**) PRV-Cherry-labeled precerebellar neurons (magenta) are in contact with dI2 axonal terminals (cyan). (**G**) Density plots of dI2 synapses and precerebellar neuron somata (density values 10–90%) in the sciatic plexus segments (N = 4735 synapses and N = 289 cells, respectively). (**H**) The laminar distribution of precerebellar neurons, dI2 neurons, and dI2 synapses at the sciatic level. See Figure 2—source data 1. Figure 2—source data 1.Localization of precerebellar neurons and dI2 synapses at the sciatic level.The X/Y coordinates of precerebellar neurons (Figure 2E) and dI2 synapses (Figure 2G) at the sciatic level.

To study the supraspinal targets of dI2 neurons, axonal and synaptic reporters were expressed in lumbar dI2 neurons (Figure 2A). At stage HH18 (E3), dI2 enhancers were co-electroporated with the double conditional axonal reporter membrane-tethered Cherry and the synaptic reporter SV2-GFP (Figure 1—figure supplement 1A). Expression in the lumbar spinal cord was attained by using thin electrodes positioned near the lumbar segments. At E17, the stage in which the internal granule layer is formed in the chick cerebellum, the axons and synapses of dI2 neurons were studied. dI2 axons cross the spinal cord at the floor plate at the segmental level, ascend to the cerebellum, enter through the superior cerebellar peduncle, and cross back to the ipsilateral side of the cerebellum (Figure 2B). Synaptic boutons were noticeable in the granule layer at the ipsilateral and contralateral sides of the anterior cerebellar lobules (Figure 2C). Synaptic boutons were also present in the central cerebellar nuclei (Figure 2—figure supplement 1A).

**Figure 2—figure supplement 1.**
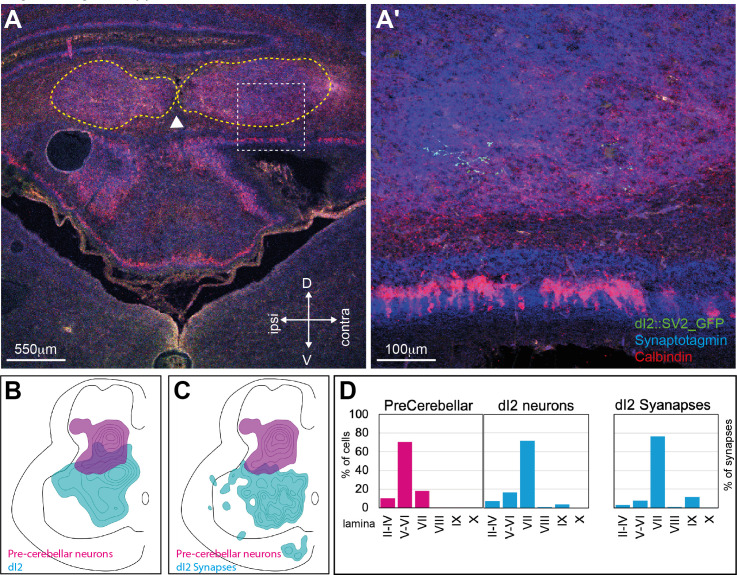
Cerebellar and central cerebellar nucleus targets of dI2 neurons. (**A**) dI2 synapses in the central cerebellar nuclei. A cross-section of an embryonic day (E) 17 chick cerebellum. dI2 synapses (magenta), synaptotagmin (cyan). The arrowhead points to the midline, the yellow dash lines encircle the central cerebellar nuclei, and the area confined in the white square is enlarged in (**A’**). (**B**) Density plot of dI2 and precerebellar neurons at the crural plexus segments (N = 551 and N = 652 cells, respectively; N = 2 embryos). (**C**) Density plot of dI2 synapses and precerebellar neurons at the crural plexus segments (N = 2543 synapses with density values of 25–80% and N = 652 cells with density values of 10–90%, respectively). (**D**) Laminar distribution of precerebellar neurons, dI2 neurons, and dI2 synapses in the different lamina at the crural level. Figure 2—figure supplement 1—source data 1.Localization of precerebellar neurons and dI2 synapses at the crural level.The X/Y coordinates of precerebellar neurons (Figure 1—figure supplement 1B) and dI2 synapses (Figure 2—figure supplement 1C) at the crural level.

The difference in soma size between dorsally and ventrally located dI2 neurons prompted us to test which dI2 neurons project to the cerebellum. dI2 neurons and precerebellar neurons were colabeled by genetic targeting of dI2 at early stages of embryogenesis (HH18), coupled with intracerebellar injection of replication-defective HSV-LacZ at E15 or PRV-Cherry (Figure 2A). Cholera toxin subunit B (CTB) was coinjected with PRV-Cherry to verify primary infection of precerebellar neurons. Spinal neurons retrogradely labeled from the cerebellum consist of double-crossed VSCT neurons and ipsilaterally projecting DSCT neurons. However, dI2 neurons, double labeled by genetic targeting and retrograde labeling from the cerebellum, are all VSCT neurons since dI2 neurons are commissural neurons. The soma distributions of precerebellar neurons, dI2 neurons, and dI2 synapses overlapped at the sciatic level (Figure 2E, G and H) and to a lesser extent at the crural level (Figure 2—figure supplement 1B–D). We found that large-diameter dI2 neurons were mostly colabeled, most of them in the ventral aspect of lamina VII (Figure 2D). Interestingly, many of the cherry^+^ and LacZ^+^ neurons were contacted by dI2 axons (Figure 2F), suggesting that dI2 neurons innervate precerebellar neurons.

**Figure 3.**
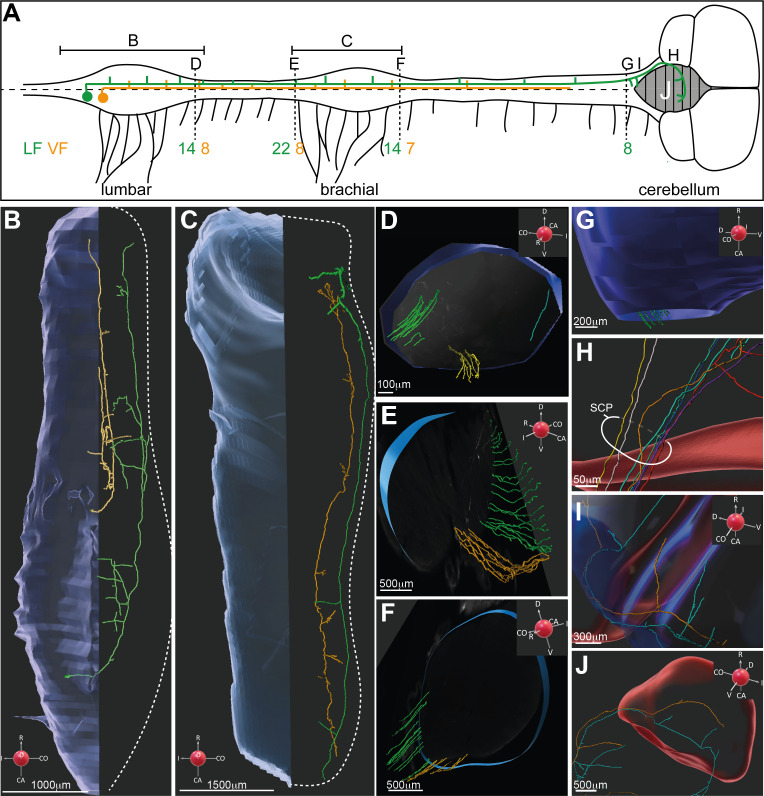
3D reconstruction of dI2 neurons along the rostrocaudal axis. (**A**) Spinal cord scheme describing dI2 axonal projection along the rostrocaudal axis (caudal is to the left, and rostral is to the right). The full lines represent the lumbar and brachial levels shown in (**B**) and (**C**). The broken lines represent the cross-sections shown in (**D–G**). The number of axons (and the funicular division) along the rostrocaudal axis is indicated adjacent to the corresponding letters (**D–G**). (**B, C**) Two representative dI2 neurons projecting their axons in the lateral funiculus (LF; green) and ventral funiculus (VF; yellow) at the lumbar (**B**) and brachial (**C**) levels. Numerous axonal collaterals are apparent. (**D–F**) Cross-sections at different levels of the spinal cord showing dI2 axons exiting the rostral end of the lumbar segments (**D**), entering the caudal brachial level (**E**), and exiting the rostral brachial level (**F**). Green: LF on the contralateral side (cLF); orange: VF on the contralateral side (cVF); cyan: LF on the ipsilateral side (iLF). (**G–J**) dI2 axons in the brainstem and cerebellum. (**G**) Axons entering the brainstem are indicated in green. (**H**) dI2 axons enter the cerebellum via the superior cerebellar peduncle (SCP). (**I**) Collaterals projecting into the brainstem. (**J**) The axons cross the cerebellar midline back to the ipsilateral side (two representative axons). A coordinate system is supplied in (**B–G**, **I, J**).

Segmental crossing at the lumbar level and recrossing back to the ipsilateral side at the cerebellum are characteristic of the VSCT projection pattern. To measure the proportion of precerebellar neurons in dI2, we used sparse labeling of lumbar dI2 neurons coupled with whole-mount imaging of the E13 spinal cord from the sacral level to the cerebellum utilizing light-sheet microscopy and iDISCO imaging (Belle et al., 2014; Renier et al., 2014; Figure 3; Video 1, Video 2, Video 3, Video 4). 85 neurons were labeled at the lumbar level, 17 of which were large-diameter neurons. The axons of all dI2 neurons cross the midline. Longitudinally projecting axons were apparent at the contralateral VF and LF (Figure 3A–F; Video 1, Video 2, Video 3). In the gray matter, axonal ramifications originating from collateral branching were apparent along the entire extent of the spinal cord (Figure 3A–C; Video 1, Video 2, Video 3). Bifurcation of the longitudinal axons was also apparent. The bifurcating branch elongated rostrally for a few segments and subsequently turned transversely into the spinal cord (Figure 3—figure supplement 1A). We counted the numbers of longitudinally projecting axons at different levels (Figure 3A and D-H): 22 axons at LS1 (Figure 3D), 30 at T1 (Figure 3E), 21 at C9 (Figure 3F), 8 at the rostral brain stem (Figure 3G), and 7 entering the cerebellum via the superior cerebellar peduncle (Figure 3H). The elevated number of axons in T1 levels likely reflects longitudinal bifurcations. Considering that the spinal cord was observed at E13, a relatively early stage of development, it is likely that additional axons enter the cerebellum at later stages of development.

**Figure 3—figure supplement 1.**
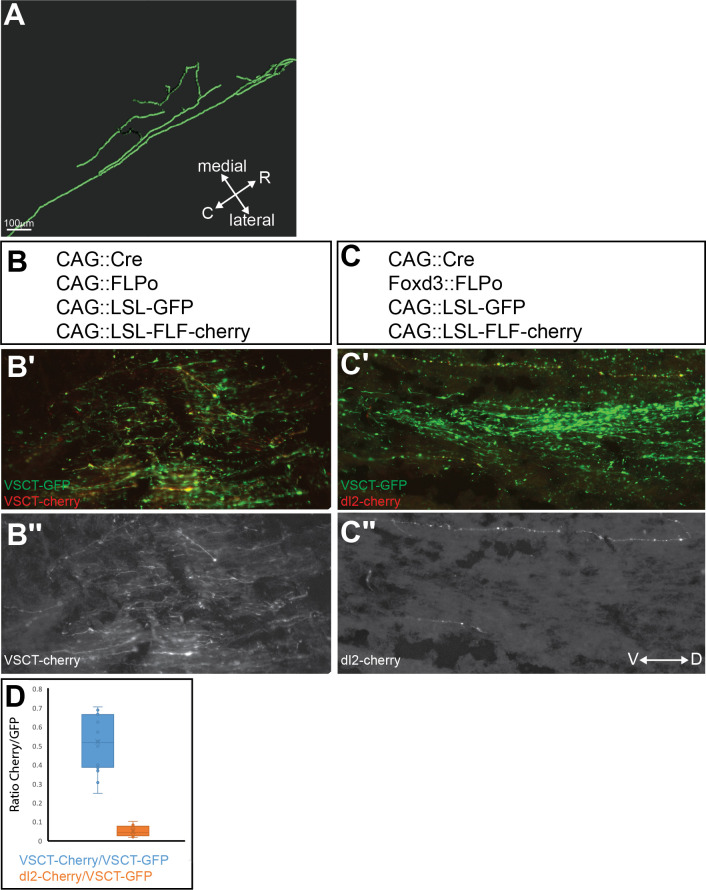
dI2 projection neurons constitute 10% of neurons in the ventral spinocerebellar tract (VSCT). (**A**) A longitudinal bifurcation of a rostrally projecting dI2 axon. 3D reconstructions of light-sheet microscopy images of a lumbar-level dI2 neuron projecting at the brachial level. The bifurcated branch initially projects rostrally and subsequently turns medially into gray matter. (**B, C**) Proportion of dI2 cells among all VSCT neurons. GFP expression was activated by the removal of a single STOP cassette via Cre recombinase, and Cherry expression was activated by the removal of two STOP cassettes via Cre and FLPo recombinases. In the control experiment (**B**), the expression of Cre and FLPo was driven by the ubiquitously expressed CAG enhancer/promoter, while expression in dI2 cells (**C**) was controlled by CAG::Cre and dI2 enhancers driving FLPo. (**B′–C′**) A section at the level of the superior cerebellar peduncle. The ratio of the number of Cherry^+^ (VSCT or dI2, in **B′, B″** and **C′, C″**, respectively) axons to the number of GFP^+^ (VSCT) axons was calculated. (**D**) Quantification of Cherry^+^/GFP^+^ in each experiment. In control electroporation, the ratio of Cherry to GFP was 0.52 ± 0.059 (blue box). The proportion of Cherry-expressing dI2 axons was 0.05 ± 0.01 (orange box). Thus, considering the 50% efficiency of double versus single conditional removal of the STOP signal, the proportion of dI2 axons was 9.61% (**B** and **C**, N = 2 and 3 embryos, respectively). See Figure 3—figure supplement 1—source data 1. Figure 3—figure supplement 1—source data 1.Analysis if the dI2 and ventral spinocerebellar tract (VSCT) axons at the superior peduncle.The number of axons expressing GFP or/and cherry at the superior peduncle (Figure 3—figure supplement 1D).

**Video 1.** dI2 interneurons (INs): transverse sections of lumbar segments. Caudal to rostral transverse images of light-sheet microscopy images along the lumbar segments of an embryonic day (E) 13 spinal cord expressing Cherry in dI2 neurons (a reference to the location of the section is shown at the bottom left). dI2 cell bodies and axons are visible. Examples of large- and small-diameter dI2 neurons are indicated by arrows. The concentration of axons on the side contralateral to electroporation is clear.

**Video 2.** dI2 interneurons (INs): lumbar segments. 3D reconstruction of light-sheet microscopy images of the lumbar spinal cord. In the transparent mode, dI2 axons are apparent on the contralateral side. The trajectory of two representative axons (ventral and lateral projection axons in yellow and green, respectively) was reconstructed. Significant branching is apparent. All the axons exiting the lumbar segments are visible. A coordinate system is supplied in key frames.

**Video 3.** dI2 interneurons (INs): brachial segments. 3D reconstruction of light-sheet microscopy images of dI2 axons entering and exiting the brachial spinal cord. Two representative axons (ventral and lateral projection axons in yellow and green, respectively) were followed, and their collaterals along the spinal cord are demonstrated. A coordinate system is supplied in key frames.

**Video 4.** dI2 interneurons (INs): brainstem and cerebellum. 3D reconstruction of light-sheet microscopy images of dI2 axons projecting into the brain stem and the cerebellum (blue and red, respectively). Cerebellum midline crossing is demonstrated for two representative axons. The axonal collaterals to the brainstem are apparent. A coordinate system is supplied in key frames.

To evaluate the complexity of the branching pattern, we reconstructed the axonal projection and branching pattern of two large-diameter dI2 neurons at the lumbar and brachial levels (Figure 3B and C). Numerous collaterals that penetrate the spinal cord along its entire length were evident. Importantly, VSCT dI2 neurons projected to spinal targets at the lumbar, thoracic, and brachial spinal levels and to the brain stem and cerebellum (Figure 3).

To measure the proportion of dI2 in VSCT neurons, we labeled VSCT axons with GFP and dI2 axons with Cherry (for experimental design, see Figure 3—figure supplement 1B and C). The number of axons expressing the reporters at the contralateral superior cerebellar peduncle was scored. 10% of the VSCT axons belonged to dI2 neurons (Figure 3—figure supplement 1D). Thus, the large-diameter dI2 neurons constitute 10% of the VSCT neurons, consistent with the anatomical observation that the VSCT comprises a heterogeneous population of INs (Jankowska and Hammar, 2013; Stecina et al., 2013).

### Mapping the synaptic input and output of dI2 neurons

To obtain the connectome of dI2 neurons, we employed enhancer-mediated synaptic labeling of presynaptic neurons coupled with soma labeling of postsynaptic neurons. We used three criteria for assessing synaptic contact: (1) the likelihood of connectivity was examined by spatial overlap of axonal terminals from the presumed presynaptic neurons and the somata of the postsynaptic neurons; (2) synaptic boutons were detected on the somatodendritic membrane of postsynaptic neuron; and (3) colabeling was observed between the presynaptic reporter and synaptotagmin (syn). We used confocal imaging and 3D reconstitution to score overlap (Figure 4—figure supplement 1A, B).

As a proof of concept, we tested the colabeling of dI2::SV2-GFP and syn in dI2 to contralateral pre-MN synapses. Of 144 genetically labeled boutons, 121 (84%) were syn^+^. The syn^-^ boutons were significantly smaller (Figure 4—figure supplement 1C). We set a volume threshold (0.07 µm^3^, Figure 4—figure supplement 1C), and small-volume SV2-GFP boutons were not considered synapses in the study. Using these criteria, we mapped the putative pre-dI2 and post-dI2 neurons.

### dI2 neurons receive synaptic input from pre-MNs and sensory neurons

**Figure 4.**
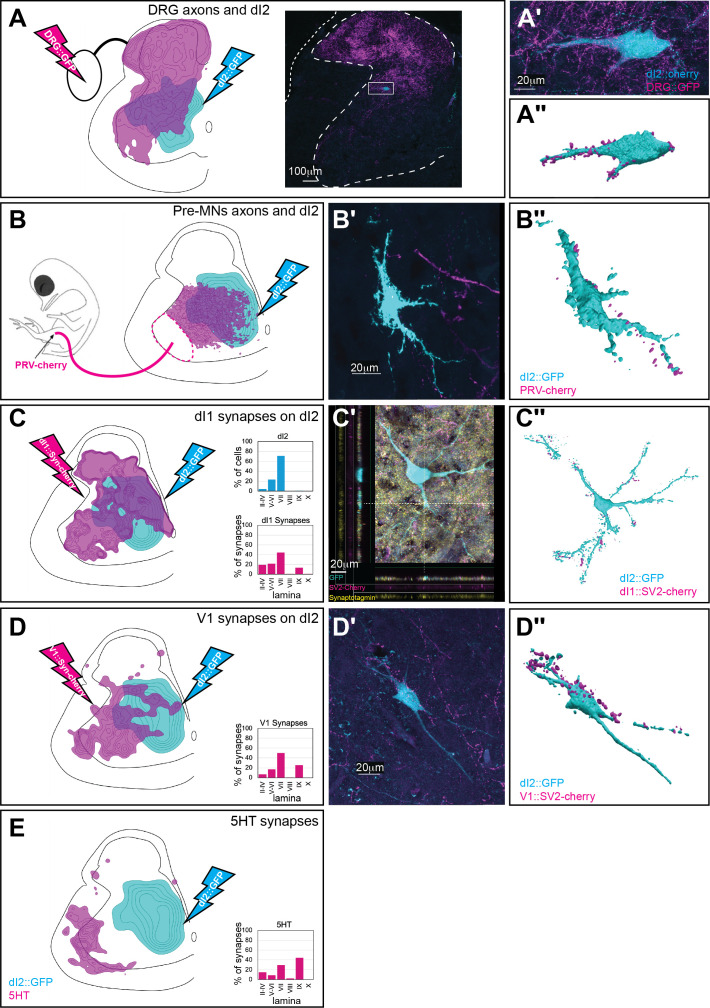
Synaptic inputs to dI2 neurons. Schematic representations of the experimental design for labeling dI2::GFP or dI2::Cherry interneurons (INs; cyan) and potential sources of synaptic inputs (magenta). The soma densities of dI2 INs and the synaptic densities are illustrated in (**A–E**). The density values presented are 10–80%, 20–80%, 25–80%, 30–50%, and 20–80%, respectively. The laminar distributions are illustrated on the right side of (**A–E**). Examples of dI2 neurons contacted by axons or synaptic boutons are shown in (**A′–D′**), and their 3D reconstruction is shown in (**A″–D″**). Genetic labeling was achieved using specific enhancers (Figure 1—figure supplement 1A) introduced by electroporation at HH18. (**A**) Dorsal root ganglion (DRG) neurons form contacts on dI2 neurons. Inset in (**A**): cross-section of embryonic day (E) 17 embryos at the crural plexus level of the lumbar cord. A dorsally located dI2 neuron contacted by numerous sensory afferents, magnified in (**A′**) and 3D-reconstructed in (**A″**) (N = 18 sections, the scheme was constructed based on one representative embryo). (**B**) Premotor neurons (pre-MNs) form contacts on dI2 neurons. dI2 neurons were labeled at HH18. At E13, PRV virus was injected into the leg musculature, and the embryo was incubated until the infection of the pre-MNs (39 hr) (N = 34 sections, the scheme was constructed based on two representative embryos). (**C**) dI1 neurons form synapses on dI2 neurons. (N = 8568 synapses, 2 embryos). (**C′**) A representative SV2::Cherry synapse on dI2 dendrites positive for synaptotagmin. Demonstrated by horizontal and vertical optical sections in the Z-axis (see cursors and color channels). (**D**) V1 neurons form synapses on dI2 neurons (N = 1923 synapses, 2 embryos). (**E**) dI2 neurons are not contacted by 5-HT synaptic terminals (N = 1718 synapses, 1 embryo). E17 cross-sections of dI2::GFP-labeled embryos were stained for 5-HT. See Figure 4—source data 1. Figure 4—source data 1.Localizations of pre-dI2 terminals and synapses at the sciatic level.The X/Y coordinates of dorsal root ganglion (DRG) axons and premotor neurons (pre-MNs) axons (Figure 4A and B). The X/Y coordinates of dI1, V1, and synapses and 5HT terminals at the sciatic level (Figure 4C–E).

To assess the synaptic input to dI2 neurons, we investigated their synaptic connectivity with the following: (1) dorsal root ganglion (DRG) neurons (Figure 4A). (2) Ipsilateral pre-MNs. General ipsilateral pre-MNs were labeled by injecting a PRV-Cherry virus into the ipsilateral hindlimb musculature (Hadas et al., 2014; Figure 4B). Two genetically defined classes of pre-MNs were examined: dI1i excitatory INs (Figure 4C, Figure 4—figure supplement 2) and the V1 inhibitory pre-MN population (Bikoff et al., 2016; Gosgnach et al., 2006; Figure 4D). (3) Reticulospinal tract neurons (Figure 4E). dI2, DRG, V1, and dI1 neurons were labeled using specific enhancers (Figure 1—figure supplement 1A).

**Figure 4—figure supplement 1.**
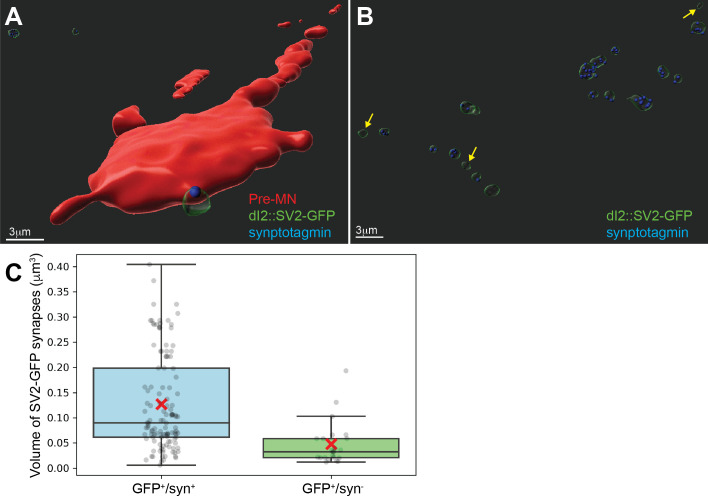
Validation of the synaptic reporter as an indicator of synapses. (**A**) Premotor neurons (red) contacted by dI2::SV2-GFP and colabeled with synaptotagmin (syn in blue) antibody. (**B**) A group of SV2-GFP-labeled boutons and the *syn* content (blue foci). Note that only the syn^-^ boutons are small SV2-GFP boutons (yellow arrows). (**C**) Quantification of the volume of GFP^+^ boutons positive or negative for *syn*. The SV2-GFP^+/^syn^+^ boutons had a larger volume than the SV2-GFP^+^syn^-^ boutons. (N = 121 and N = 23 for syn-positive and -negative GFP^+^ synapses, respectively, from one representative embryo. p<0.001, t-test). Figure 4—figure supplement 1—source data 1.Validation of the use of SV2-GFP reporter as an indicator for synapses.The volume of GFP^+^/syn^+^ and GFP^+^/syn^-^ dI2 synapses on premotor neurons (Figure 4—figure supplement 1C).

**Figure 4—figure supplement 2.**
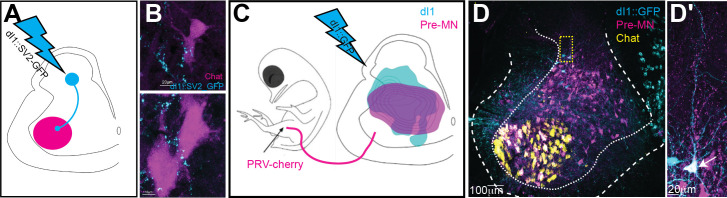
dI1i neurons are premotor neurons (pre-MNs). (**A, B**) Schematic illustration of the strategy for studying the potential innervation of motor neurons by dI1 neurons (**A**). dI1 neurons were targeted by a dI1-specific enhancer (Figure 1—figure supplement 1A). At embryonic day (E) 15, synaptic boutons labeled with SV2-GFP were studied on cross-sections. A synaptic reporter (cyan) was expressed in dI1 neurons. dI1i boutons contacting Chat^+^ motor neurons (magenta) are apparent (**B**). (**C, D**) Schematic representation of the experimental design for colabeling dI1 and pre-MNs, supplemented by cell soma densities of dI1 neurons (cyan, N = 643 cells) and pre-MNs (magenta, N = 936 cells). dI1 neurons were labeled at HH18. At E13, PRV virus was injected into the leg musculature, and the embryo was incubated until the infection of the pre-MNs (35 hr) (**C**). In a cross-section taken at E13, PRV-Cherry (magenta) was detected in motor neurons (Chat^+^, yellow) and pre-MNs (**D**). An example of dI1 neurons coexpressing GFP and PRV-Cherry is shown in (**D′**). Figure 4—figure supplement 2—source data 1.Localization of dI1 synapses.The X/Y coordinates of dI1 synapses at the sciatic level (Figure 4—figure supplement 2C).

**Figure 4—figure supplement 3.**
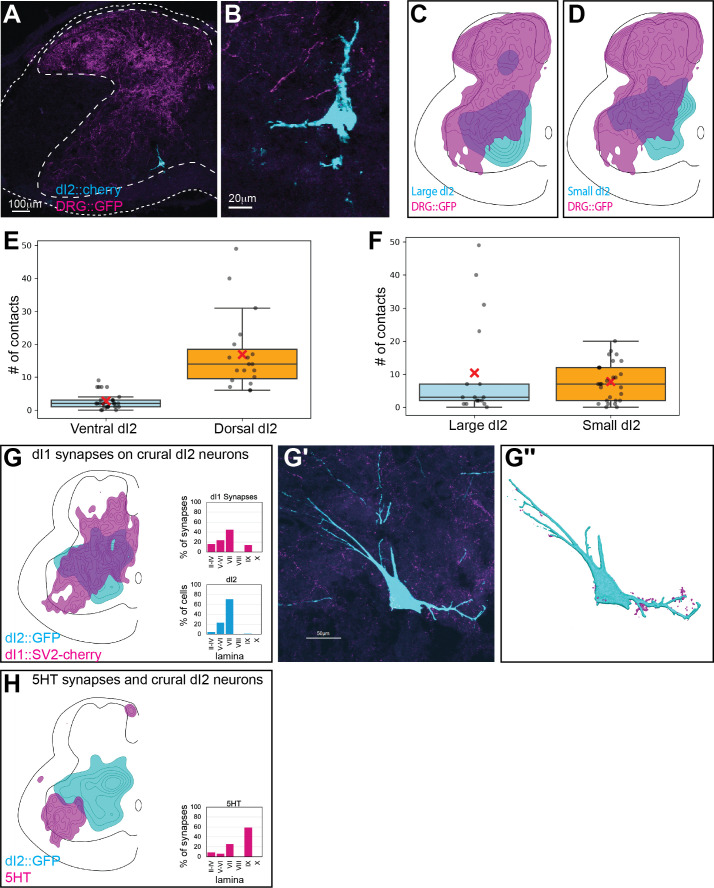
Input of dorsal root ganglion (DRG), dI1, and 5-HT neurons to dI2 neurons at the level of the crural plexus. (**A, B**) Sparse sensory innervation of ventrally located dI2 neurons. Cross-section of an embryonic day (E) 17 embryo at the lumbar spinal cord (crural plexus level). A ventrally located dI2 neuron is sparsely contacted by sensory afferents (**A**), magnified in (**B**). (**C, D**) Density plots of dI2_large_ (magenta, N = 48) and dI2_small_ (yellow, N = 502) neurons and sensory afferents (N = 18 sections, with density values 10–80%) in the crural plexus segments. (**E, F**) Quantification of the number of contacts between terminals of DRG axons and dI2 neurons. The number of contacts was significantly higher in dorsal than ventral dI2 neurons (**E**) (N = 26 and N = 19 for ventral and dorsal neurons, respectively). p<0.00001, t-test. There was no significant difference in the number of contacts on either small or large dI2 neurons (**F**) (N = 17 and N = 28 for large and small neurons, respectively). p=0.49, t-test. (**G**) dI1 neurons form synapses on dI2 neurons. Density plot and the laminar distribution of dI1 synapses (magenta) and dI2 somata (cyan) (N = 13,369 synapses with density values 50–90%). Example of dI1 boutons (magenta) on a dI2 neuron (cyan) (**G′**) and its 3D reconstruction in (**G″**). (**H**) dI2 neurons are not contacted by 5-HT synaptic terminals. Density plot and laminar distribution of 5-HT synapses (magenta) and dI2 somata (cyan) (N = 2754 synapses with density values 25–85%). Figure 4—figure supplement 3—source data 1.Distribution of dorsal root ganglion (DRG) terminals on dI2 neurons.The ventral/dorsal location and the large/small somata of dI2 neurons that are contacted by DRG terminals (Figure 4—figure supplement 3E,F). Figure 4—figure supplement 3—source data 2.Localizations of pre-dI2 terminals and synapses at the crural level.The X/Y coordinates of dI1 synapses and 5HT terminals at the crural level (Figure 4—figure supplement 3G,H).

A density profile of the axons of DRG neurons (Figure 4A, Figure 4—figure supplement 3A–D) was aligned with the density plots of the dl2 somata. Overlap between the axonal terminals of DRG neurons was evident (Figure 4A, Figure 4—figure supplement 3C and D). Contact between DRG axons and dI2 neurons was mainly apparent in the dorsal dI2 neurons, while the ventral dI2 neurons received little to no input from DRG neurons (2.8 ± 2.4 vs. 16.9 ± 11.3 contacts per neuron for ventral and dorsal dI2 neurons, respectively; p<1e-5; Figure 4A, Figure 4—figure supplement 3E). In contrast, large- and small-diameter dI2 neurons did not exhibit a significant difference in DRG axon contacts (10.4 ± 14.9 vs. 7.8 ± 5.7 contacts per neuron for large and small dI2 neurons, respectively; p=0.4) (Figure 4—figure supplement 3F).

The density plot of PRV-labeled pre-MNs overlapped with the density plots of the dl2 somata (Figure 4B), and the axonal terminals of pre-MNs were visible on dI2 somata and dendrites (Figure 4B′,B′′), suggesting that pre-MNs contacted dI2 neurons. To solidify the evidence for pre-MN/dI2 connectivity, we used synaptic reporters expressed in genetically identified dI1 and V1 pre-MNs. Excitatory dI1 synapses and inhibitory V1 synapses overlapped with the density plots of the dl2 somata (Figure 4C and D). Synaptic connections, evaluated by boutons found on dI2 dendrites and somata, were apparent from V1 and dI1i (Figure 4B–D, Figure 4—figure supplement 3G).

Serotonergic neurons are the main reticulospinal input to VSCT in cat (Hammar et al., 2004; Hammar and Maxwell, 2002). Serotonergic synapses were concentrated on motor neurons and were not observed on dI2 neurons (Figure 4E, Figure 4—figure supplement 3H). Double labeling of 5-HT and dI2 neurons did not reveal any synaptic input. The lack of synaptic serotonergic input may be related to the difference in species or may suggest that other, non-dI2 VSCT neurons located adjacent to motoneurons are contacted by the reticulospinal neurons. The analysis of synaptic inputs supports the concept that dI2 neurons constitute part of the VSCT. These cells receive input from sensory afferents and inhibitory and excitatory pre-MNs and project to the cerebellum.

### dI2 neurons innervate contralateral lumbar and brachial pre-MNs and dI2 neurons

**Figure 5.**
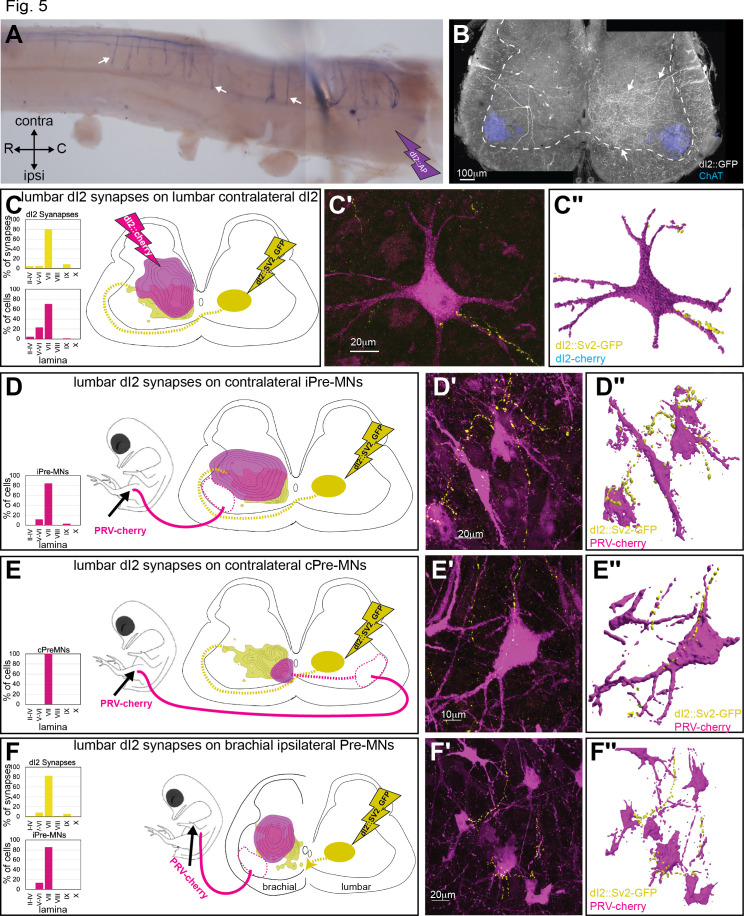
Spinal synaptic targets of dI2 neurons. (**A**) Whole-mount staining of the spinal cord (thoracic segments) expressing alkaline phosphatase (AP) in dI2 neurons. The lumbar dI2 neurons (not included in the image) were labeled with AP. dI2 axon collaterals project and into the spinal cord (arrows). Abbreviations in the coordinates: rostral: R: caudal: C. (**B**) Cross-section of an embryonic day (E) 17 embryo at the crural plexus level of the lumbar spinal cord. Axonal collaterals (white arrow) penetrating the gray matter of the contralateral side are evident. Schematic representations of the experimental design for labeling synapses (dI2::SV2-GFP, yellow) and potential targets (magenta) supplemented by cell soma density and dI2 synaptic densities are illustrated in (**C–F**). The laminar distribution of the somata and synapses is illustrated on the right side of (**C–F**). Examples of target neurons contacting synaptic boutons of dl2 neurons are shown in (**C′–F′**), and their 3D reconstruction is shown in (**C″–F″**). Genetic labeling was achieved using dI2 enhancers (Figure 1—figure supplement 1A) electroporated at HH18. Premotor neurons (pre-MNs) were labeled by injection of PRV-Cherry into the hindlimbs (**D, E**) or the forelimb (**F**) musculature at E13. The embryos were incubated until the pre-MNs were infected (39 hr). (**C**) dI2 neurons innervate contralateral dI2 neurons (N = 4735 synapses and N = 374 cells, respectively, two embryos). (**D**) dI2 neurons innervate ipsilateral projections of pre-MNs at the sciatic plexus level (N = 4735 synapses and N = 936 cells, respectively, scheme was done based on one representative embryo). (**E**) dI2 neurons innervate contralaterally projecting pre-MNs at the sciatic plexus level (N = 4735 synapses and N = 47 cells, respectively, scheme was done based on one representative embryo). (**F**) dI2 neurons innervate ipsilaterally projecting pre-MNs at the brachial level (N = 2215 synapses and N = 286 cells, respectively, three embryos). See Figure 5—source data 1*.* Figure 5—source data 1.Localization of dI2 synapses on post-dI2 neurons at the sciatic and brachial levels.The X/Y coordinates of dI2 synapses, ipsi premotor neurons (pre-MNs), commissural pre-MNs, and contralateral dI2 at the sciatic level; dI2 synapses and ipsi pre-MNs at the brachial level (Figure 5).

Axon collaterals of dI2 invade the gray matter along the entire length of the spinal cord, as revealed by whole-mount staining of spinal cords electroporated with an alkaline phosphatase reporter (dI2::AP) (Figure 5A), cross-sections of dI2 neurons expressing membrane-tethered EGFP (Figure 5B), and light-sheet microscopy analysis (Figure 3—figure supplement 1; Video 1, Video 2, Video 3). The region innervated by dI2 collaterals (arrow in Figure 5B) overlaps with that of the V0 and V1 pre-MNs (Lai et al., 2016; Griener et al., 2015) as well as with that of the contralateral dI2 neurons (Figures 1 and 5B). To assess the potential spinal targets of dI2 neurons, we inspected the degree of overlap between dI2 synapses and dI2 somata (Figure 5C), ipsilateral pre-MNs (Figure 5D), and contralateral pre-MNs (Figure 5E). The alignment revealed an overlap of dI2 synapses with ipsilateral/contralateral pre-MNs and dI2 neurons (Figure 5C–E), supporting their potential connectivity. Labeling of dI2 synapses coupled with labeling of the above neuronal population showed dI2 synaptic boutons on pre-MNs and dI2 neurons at the lumbar level (Figure 5C–E, Figure 5—figure supplement 1A–C).

**Figure 5—figure supplement 1.**
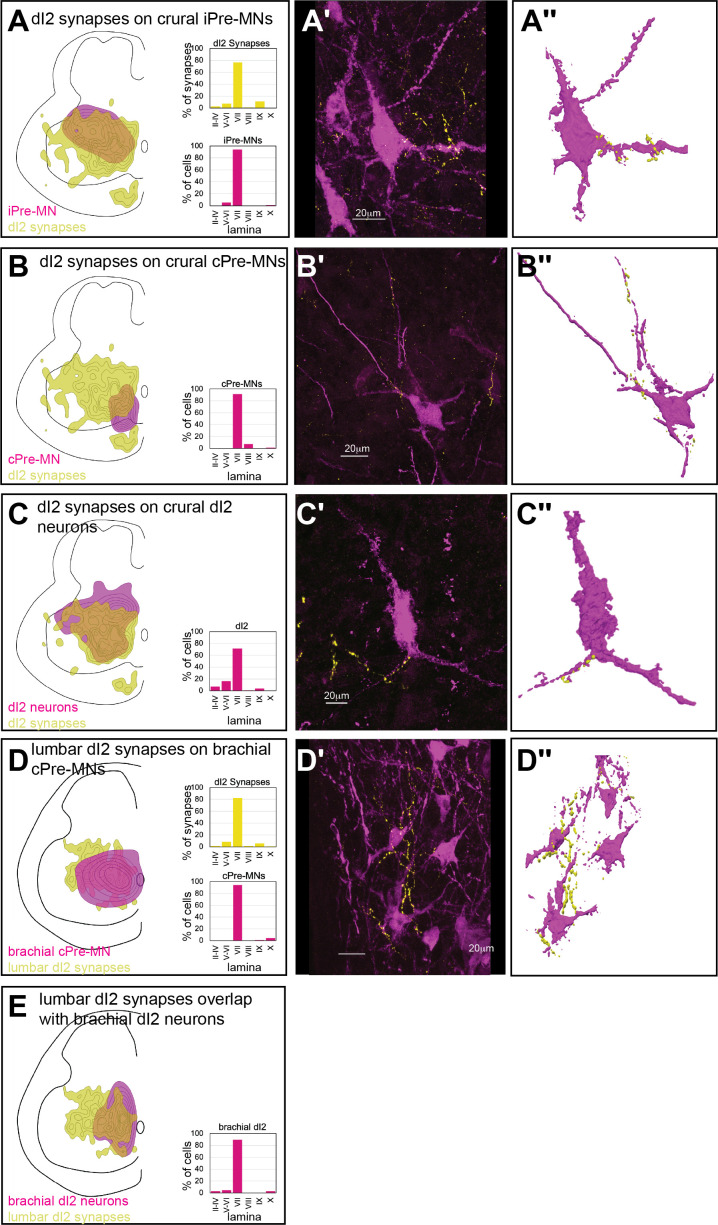
Spinal targets of dI2 at the crural and brachial levels. Cell soma density of putative targets (magenta) of dI2 neurons and dI2 synaptic densities (dI2::SV2-GFP, yellow) are illustrated in (**A–E**). The laminar distribution of the somata and synapses is illustrated on the right side of (**A–E**). Examples of target neurons in contact with dI2 synaptic boutons are presented in (**A’–E′**), and their 3D reconstruction is presented in (**A″–E″**). Genetic labeling was achieved using dI2 enhancers (Figure 1—figure supplement 1A) delivered by electroporation at HH18. Premotor neurons (pre-MNs) were labeled by injection of PRV-Cherry into the hindlimbs (**D, E**) or the forelimb (**F**) musculature at embryonic day (E) 13. Embryos were incubated for 39 hr after PRV injection. (**A**) dI2 neurons innervate ipsilaterally projecting pre-MNs at the crural plexus level (N = 2543 synapses [three embryos] and N = 250 cells, respectively, scheme was done based on one representative embryo). (**B**) dI2 neurons innervate contralaterally projecting premotor neurons at the crural plexus level (N = 2543 synapses [three embryos] and N = 117 cells, respectively, scheme was done based on one representative embryo). (**C**) dI2 neurons innervate contralateral dI2 neurons at the crural plexus level (N = 2543 synapses and N = 551 cells, respectively, two embryos). (**D**) dI2 neurons innervate brachial contralaterally projecting pre-MNs (N = 2215 synapses and N = 90 cells, respectively, two embryos). (**E**) dI2 neurons innervate brachial dI2 neurons (N = 2215 synapses and N = 66 cells, respectively, scheme was done based on two embryos).

The pattern of dI2 collaterals along the entire rostrocaudal axis (Figure 3A–C, Figure 5A) suggests that dI2 neurons innervate contralateral pre-MNs and dI2 neurons at multiple levels. To test this hypothesis, labeling of lumbar dI2 neurons was coupled with labeling of brachial pre-MNs and dI2 somata by injecting PRV into the wing musculature or electroporating a reporter into brachial dI2 neurons, respectively (Figure 5F, Figure 5—figure supplement 1D). dI2 synapses overlapped with the putative targets, and synaptic boutons originating from lumbar-level dI2 neurons were apparent on dI2 neurons and on the contralateral and ipsilateral pre-MNs of the wings (Figure 5F, Figure 5—figure supplement 1D and E).

Neuronal and synaptic labeling experiments showed that lumbar dI2 neurons innervate the cerebellum, lumbar and brachial pre-MNs, and contralateral dI2 neurons. Hence, dI2 neurons may relay peripheral and intraspinal information to the cerebellum and to the contralateral lumbar and brachial motor control centers.

### Silencing of dI2 neurons impairs the stability of bipedal stepping

The synaptic input to dI2 neurons and their putative targets implicates them as relaying information about motor activity to the contralateral spinal cord and the cerebellum. Thus, we hypothesized that manipulation of their neuronal activity may affect the dynamic profile of stepping. To study the physiological role of dI2 neurons, we silenced their activity by expressing the tetanus toxin (*TeTX*) light chain gene, which blocks synaptic transmission (Yamamoto et al., 2003), in the bilateral lumbar dI2 neurons. EGFP was cotargeted in a 2/1 TeTX/EGFP ratio for post hoc analysis of the efficacy of electroporation (Supplementary file 1). Chicks expressing EGFP in dI2 neurons and chicks that did not undergo electroporation were used as controls. To maximize the number of targeted dI2 neurons, we combined genetic targeting with the *Foxd3* enhancer and spatial placement of the electrodes at the dorsal lumbar spinal cord (Figure 1—figure supplement 1A). Embryos were electroporated at HH18. Upon hatching, chicks were trained for targeted overground locomotion. The gait parameters of four controls and 5 *TeTX*-treated chicks were measured while chicks were walking toward their imprinting trainer along a horizontal track (6–20 walking sessions, 5–8 strides each, per chick).

To test whether silencing of dI2 neurons impairs posthatching development and muscle strength, the chicks were weighed at P8, and their foot grip strength was evaluated on the same day. All chicks were of comparable weight (average – 144.7 ± 12.1 g; Supplementary file 1). As a functional measure of foot grip, we tested the ability of the chicks to maintain balance on a tilted mesh surface. *TeTX*-manipulated chicks and control chicks maintained balance on the tilted surface up to 63–70°, with no apparent statistically significant differences (Supplementary file 1). Thus, manipulation of dI2 neuronal activity did not impair the development or balance or muscle strength.

**Figure 6.**
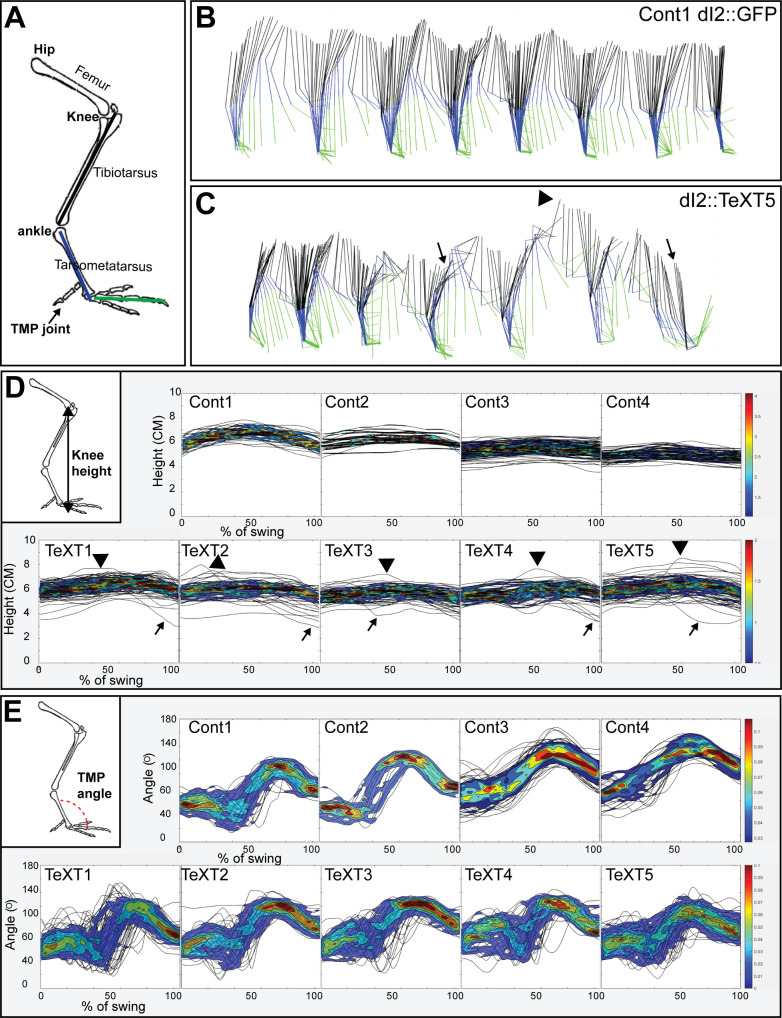
Kinematic analysis of locomotion in posthatching chicks following the silencing of dI2 neurons. (**A**) Schematic illustration of chick hindlimb joints (bold) and bones (regular). The knee joint connects the femur and the tibiotarsus, and the ankle connects the tibiotarsus and the tarsometatarsus, which connects to the phalanges at the TMP joint. During the swing phase of birds, ankle flexion leads to foot elevation, while the knee is relatively stable. (**B, C**) Stick diagrams of stepping in a control chicken d2::GFP (**B**) and in a d2::TeTX chicken (**C**). Arrows indicate collapses, and overshoots are denoted by arrowheads. (**D**) Overlays of knee height (demonstrated in insert) trajectories during the swing phase in all analyzed steps of each of the control and *TeTX*-treated posthatching day (P) 8 hatchlings are shown superimposed with the respective 20–80% color-coded density plots as a function of the percentage of swing (see text and Materials and methods). Arrows indicate collapses, and overshoots are indicated by arrowheads. (**E**) The angular trajectories of the TMP joint (shown in insert) during the swing phase in all analyzed strides of each of the control and *TeTX*-treated P8 hatchlings are shown superimposed on the respective 20–80% color coded density plots as a function of the percentage of swing (see text and Materials and methods). See Figure 6—source data 1, Figure 6—source data 2, Figure 6—source data 3. Figure 6—source data 1.Analysis of knee height trajectories during the swing phase.The knee height trajectories during the normalized swing in all the analyzed steps of all chicks (Figure 6D). Figure 6—source data 2.Analysis of TMP angles during the swing phase.The TMP angle trajectories during the normalized swing in all the analyzed steps of all chicks (Figure 6E). Figure 6—source data 3.Statistical analysis of knee height trajectories and TMP angles.Statistical analysis for the data presented in Figure 6.

**Figure 7.**
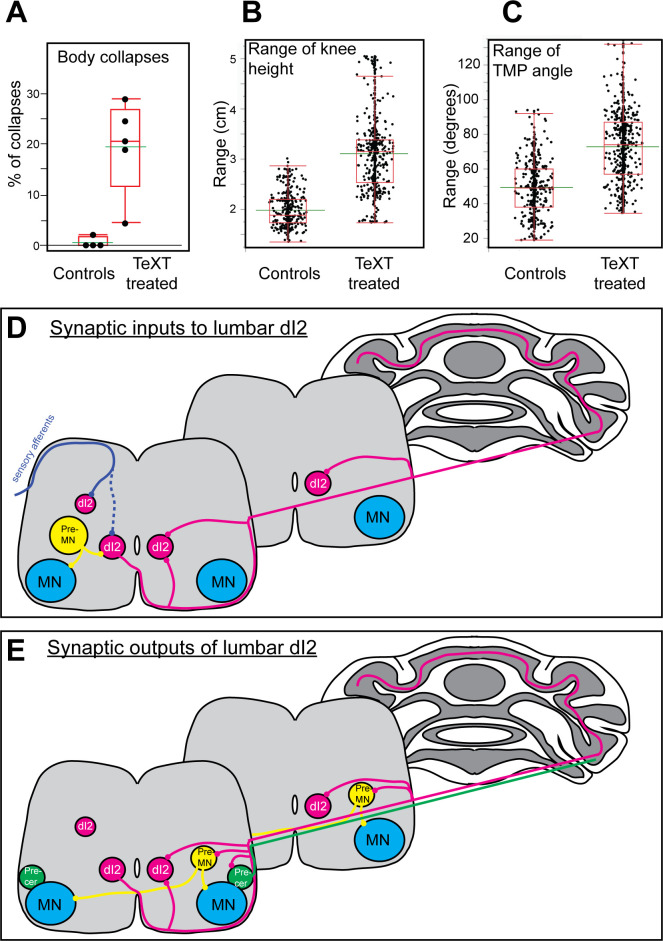
Parameters of reduced stability in bipedal stepping in *TeTX*-treated chicks. (**A**) The percentage of steps with body collapses in the controls and *TeTX*-manipulated hatchlings (n = 4 and n = 5, respectively). p-value<0.0001 (Z-test). See Table 3 for the proportions of falls at the individual chick level. (**B**) Analysis of the mean range of knee height changes during the swing phase of control and TeTX-treated chicks (n = 4 and n = 5, respectively). p-value<0.0001 using a t-test allowing different variances. See Figure 7—figure supplement 1A and Table 3 for individual chick data and statistical analysis details. (**C**) Analysis of the mean range of TMP angular excursions during the swing phase of control- and TeTX-treated chicks (n = 4 and n = 5, respectively). p-value<0.0001, Watson–Williams test. See Figure 7—figure supplement 1B and Table 3 for individual chick data and statistical analysis details. (**D, E**) Schematic illustrations showing the connectome of lumbar dI2 neurons. The synaptic inputs (**D**) and outputs (**E**) of dI2 neurons are illustrated. dI2 neurons (magenta) receive synaptic input from sensory afferents (solid blue line indicates massive synaptic input, and dashed blue line indicates sparse innervation), from inhibitory and excitatory premotor neurons (pre-MNs; yellow), and from the contralateral lumbar dI2 neurons. dI2 neurons innervate the contralateral lumbar and brachial pre-MNs (both commissural and ipsilaterally projecting pre-MNs are innervated by dI2 cells), the lumbar and brachial contralateral dI2 cells, lumbar precerebellar neurons (green), and the cerebellar granule cells. See Figure 7—source data 1. Figure 7—source data 1.Analysis of collapses.The number of collapses of each chick (Figure 7A).

Analysis of overground locomotion in the control and *TeTX*-treated chicks revealed no significant differences in swing velocity or striding pattern. A 180° out-of-phase pattern was found during stepping in all the manipulated and control chicks (Figure 6—figure supplement 1A, Table 1). However, substantial differences were scored in stability parameters: *TeTX* chicks exhibited whole-body collapses during stepping (Figures 6B and C and 7A), a wide-base gait (Table 2), and variable limb movements (Figure 6A, D and E; Figure 7B and C; Figure 7—figure supplement 1; Table 3).

**Figure 6—figure supplement 1.**
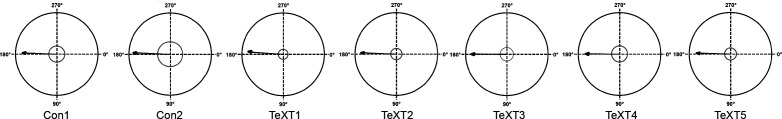
Locomotion characteristics of control and *TeTX*-treated chicks: The left-right phase. The mean left-right phase of the control and *TeTX*-treated chicks. The mean phase values are indicated by the r-vectors (arrows). The Rayleigh critical values (p=0.05) are indicated by blue circles. Figure 6—figure supplement 1—source data 1.Analysis of left-right phase.The left-right phase of the control and TeTX-treated chicks (Figure 6—figure supplement 1).

**Figure 7—figure supplement 1.**
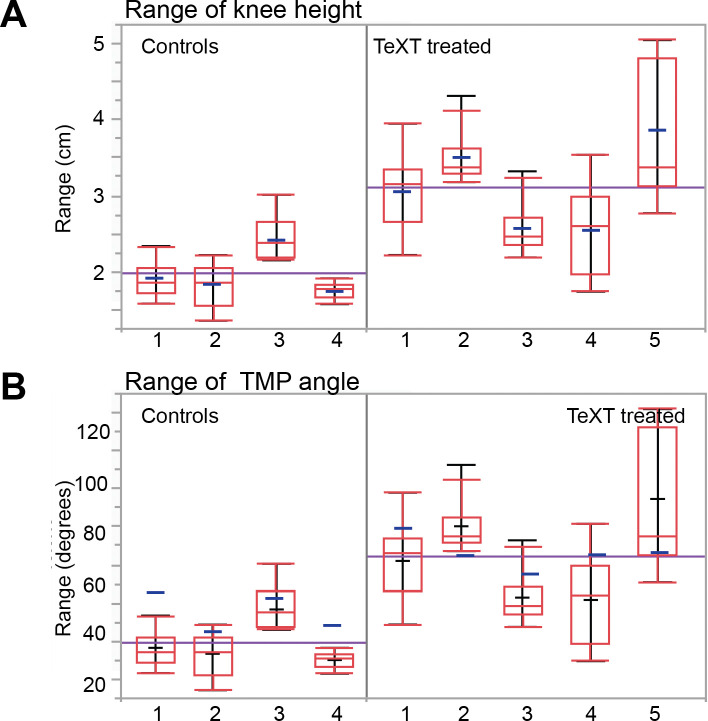
Locomotion characteristics of control and *TeTX*-treated chicks: The range of knee height and TMP angles. (**A, B**) The range of knee height and TMP angle (**A** and **B**, respectively, see text for details) for each chicken (four controls and five TeTX-treated chicks). Purple lines: averages of each group (controls and TeTX-treated chick); blue lines: averages of individual chicks; red lines and boxes: box plots; black lines: ranges of individual chicks. In general, higher interstride variability was evident in the *TeTX*-treated chicks than in the controls. Figure 7—figure supplement 1—source data 1.Statistical analysis of knee height trajectories and TMP angles of each chick.Statistical analysis for the data presented in Figure 7—figure supplement 1.

**Figure 7—figure supplement 2.**
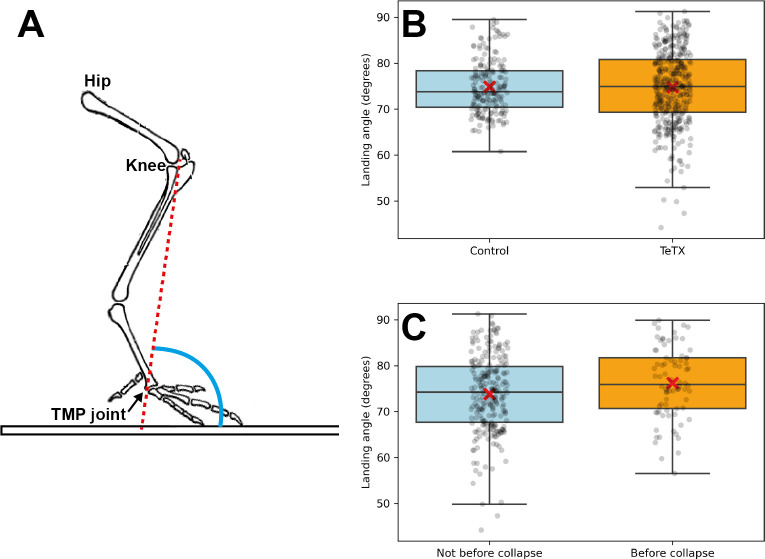
Locomotion characteristics of control and *TeTX*-treated chicks. Analyses of the landing angle. (**A**) Scheme of bones and joints of the chicken leg, indicating the landing angle. (**B**) A box plot showing the landing angles. The control and manipulated chickens did not show a significantly different landing angle (N = 188 and N = 385 steps for four control chickens and five TeTX-manipulated chickens, respectively; p=0.85, t-test). (**C**) The landing angles before collapse are slightly higher than the angles that do not precede a collapse (N = 78 and N = 248 steps for angles preceding and not preceding collapse, respectively; five chickens; p=0.02, t-test). Figure 7—figure supplement 2—source data 1.Analysis of the landing angles in all steps.The landing angles of each chick (Figure 7—figure supplement 2B). Figure 7—figure supplement 2—source data 2.Analysis of the landing angles prior to a collapse.The landing angles before and not before a collapse in each chick (Figure 7—figure supplement 2C).

**Table 1.** Stride velocity and left-right phase in control and tetanus toxin (TeTX-manipulated chicks).

Chick	Mean swing velocity (cm/s)	Mean left-right phase (°)	# of steps
TeTX1	46.78 ± 22.13	184.679 ± 33.003	113
TeTX2	62.24 ± 20.17	182.293 ± 32.01	63
TeTX3	48.06 ± 20.04	180.784 ± 31.064	69
TeTX4	57.24 ± 24.35	180.502 ± 36.291	59
TeTX5	36.66 ± 17.61	181.97 ± 35.787	93
Control 1 (GFP)	79.65 ± 37.77	182.369 ± 35.366	47
Control 2 (GFP)	41.91 ± 20.41	182.384 ± 26.708	19
Control 3 (not electroporated)	41.09 ± 16.59	N.D.	121
Control 4 (not electroporated)	42.3 ± 30.91	N.D.	51

**Table 2.** Maximum stride width in control and tetanus toxin (TeTX)-manipulated chicks.

Chick	Maximum stride width (cm)	# of steps
TeTX1	5.11 ± 1.89	97
TeTX2	5.32 ± 1.38	36
TeTX3	4.5 ± 1.01	27
TeTX4	4.9 ± 1.16	49
TeTX5	5.82 ± 1.71	110
Control 8	4.15 ± 1.07	137
Control 9	4.32 ± 1.32	115

**Table 3.** Collapses, knee height, and TMP angle ranges in control and tetanus toxin (TeTX)-manipulated chicks.

Chick	% of steps with collapse	TMP angle: mean range (°)				# of steps				% of steps with collapse
		**All steps, mean**	**Combined mean**	**Minus collapses, mean**	**Minus collapses, combined mean**	**All steps, mean**	**Combined mean**	**Minus collapses**	**Minus collapses, combined mean**	
TeTX1	4.4	3.05 ± 0.49	3.11 ± 0.74	2.87 ± 0.38	2.83 ± 0.57	82.27 ± 22	72.71 ± 20.52	79.4 ± 24.5	68.97 ± 21.47	113
TeTX2	20.60	3.5 ± 0.3		3.35 ± 0.47		71.79 ± 25		71.1 ± 25.68		63
TeTX3	18.8	2.57 ± 0.31		2.27 ± 0.26		64.17 ± 21		62.79 ± 20.9		69
TeTX4	20.45	2.54 ± 0.55		2.477 ± 0.56		72.48 ± 17		66.22 ± 19.1		59
TeTX5	29	3.86 ± 0.83		3.2 ± 0.2		72.86 ± 12		65.85 ± 13.13		93
Control 1 (GFP)	2.12	1.91 ± 0.22	1.98 ± 0.33	1.91 ± 0.22	1.98 ± 0.33	56.8 ± 16.4	49.34 ± 16.03	56.95 ± 16.6	49.4 ± 16.18	47
Control 2 (GFP)	0	1.83 ± 0.25		1.83 ± 0.25		41.42 ± 18.4		41.42 ± 18.4		19
Control 3 (not electroporated)	0	2.42 ± 0.23		2.42 ± 0.23		54.86 ± 9.65		54.86 ± 9.65		121
Control 4 (not electroporated)	0	1.75 ± 0.1		1.75 ± 0.1		44.12 ± 12.51		44.12 ± 12.51		51

#### Whole-body collapses

A collapse was scored as a decline in knee height below 85% of the average knee height at the stance phase of the step (arrow in Figure 6C). We measured the number of collapses in 50–190 steps. In control chicks, collapses occurred in 0.53% ± 0.92% of the steps. In *TeTX*-manipulated chicks, we observed collapses in 19.46% ± 8.3% of the steps, which was significantly different from the rate in controls (Figure 7A). As some collapses were followed by an overextension (‘overshoot’ in leg elevation), also manifested in the profile of the knee height trajectory during the swing phase (Figure 6D), we further studied the relationship between the two phenomena. In general, there was high variability between chicks in this aspect.

Most collapses (64.9% ± 19.7%) were not preceded or followed by overextension. About 22% of the collapses (22.47% ± 21.5% of collapses, e.g., arrowhead in Figure 6C) were followed by overextension, suggesting a postcollapse compensation in the extensor drive. The rest of the collapses (12.63% ± 7.56%) were preceded by overextension.

#### Wide-base stepping

A wide-base stance is typical of an unbalanced ataxic gait. The stride width was measured between the two feet during the double stance phase of stepping. The mean stride in *TeTX*-manipulated chicks 1, 2, 4, and 5 was significantly wider than that in the control chicks, while the width in *TeTX3* was similar to that in the controls (Table 2).

#### Variable limb movements

In stable gait, limb trajectories are consistent from stride to stride. Therefore, we compared the trajectories of knee height and angle of the TMP joint during the swing phase of stepping between control and TeTX-manipulated chicks. Plots of the knee height and TMP angle trajectories during the normalized swing in all the analyzed steps of each chick are shown superimposed in Figure 6D and E, respectively. These data demonstrate that the range of changes in *TeTX*-manipulated chicks was higher than that in control chicks.

Further analyses revealed that, overall, the control group showed lower knee height and TMP angle ranges than the TeTX-treated group, even though there were differences within groups (Figure 7—figure supplement 1). The average knee height range of the combined control chicks (1.981 ± 0.33) was significantly lower than the range of the combined TeTX-treated chicks (3.109 ± 0.74) (Figure 7B). A similar comparison of the combined ranges of angular excursions of the TMP joint during the normalized swing revealed that the average angle of the control group (49.34 ± 16.03) was significantly lower than the average of the TeTX-treated chicks (77 ± 22.15; Figure 7C).

Since the increased range of changes could be due to the effects of the substantial increase in body collapses during stepping (Figure 7A, see also Figure 6), we excluded steps featuring whole-body collapses and reanalyzed the data. The data summarized in Table 3 show that the significant difference between controls and the *TeTX*-treated chicks in the range of the knee height and the TMP angle excursions was maintained. Thus, the increase in irregularity in the *TeTX*-treated chicks is not caused exclusively by the body collapses of the *TeTX*-treated chicks.

Loss of balance can also arise from slipping, which can originate from a shallow landing angle (Clark and Higham, 2011). The landing angle was characterized as the angle between the ground and the imaginary line connecting the knee joint (which is located near the chicken’s center of mass; Daley and Biewener, 2006) to the TMP joint at the end of the swing phase (Figure 7—figure supplement 2A). Thus, we analyzed the landing angles of the manipulated and control chickens. No significant differences in landing angles were detected (Figure 7—figure supplement 2B). Additionally, we compared the landing angle before a collapse to landing angles not preceding a collapse. We found that the angles preceding a collapse were not smaller and even tended to be slightly larger than the landing angles that did not precede a collapse (Figure 7—figure supplement 2C, p=0.02). These results argue against the possibility that the increased frequency of collapses in the manipulated chickens stemmed from slipping and sliding events.

Overall, the kinematic parameters of the dI2-TeTX-treated chicks demonstrate a reduction in stability during locomotion, indicating a possible role of dI2 in the stabilization of bipedal stepping.

## Discussion

The VSCT is thought to provide peripheral and intrinsic spinal information to the cerebellum to shape and update the output of spinal networks that execute motor behavior. The lack of genetic access to VSCT neurons hampers efforts to elucidate their role in locomotion. Using a genetic toolbox to dissect the circuitry and manipulate neuronal activity in the chick spinal cord, we studied spinal INs with VSCT characteristics. The main finding in our study is that dI2 neurons in the chick lumbar spinal cord are commissural neurons that innervate pre-MNs at the contralateral lumbar and brachial spinal levels and granule neurons in the ipsilateral cerebellum. Hence, a subpopulation of dI2 neurons form part of the avian VSCT. Targeted silencing of dI2 neurons leads to impaired stepping in P8 hatchlings. We described the spatial distribution of subpopulations of dI2 neurons, deciphered their connectomes, mapped the trajectory of their projections to the cerebellum, and suggested possible mechanisms for the gait perturbation resulting from their genetic silencing, as discussed below.

### The connectome of dI2 neurons

Using the intersection between genetic drivers and spatially restricted delivery of reporters to define lumbar and brachial neurons, we identified several targets of dI2 lumbar neurons. Lumbar dI2 neurons innervate contralateral lumbar dI2 neurons as well as commissural and noncommissural lumbar pre-MNs. This connectivity may influence the bilateral spinal output circuitry at the lumbar cord (e.g., Bras et al., 1988; Jankowska and Hammar, 2013). Moreover, the ascending axons of lumbar dI2 neurons give off gray matter collaterals innervating contralateral dI2 neurons and commissural and noncommissural pre-MNs throughout the brachial spinal cord (Figure 7D and E). Therefore, lumbar dI2 neurons may also contribute to the inter-enlargement coupling described between the segments of the spinal cord that move the legs and the wings (e.g., Valenzuela et al., 1990; Ruder et al., 2016 for forelimb to hindlimb coupling connectivity in mice).

We demonstrated that lumbar dI2 cells receive sensory innervation, premotor inhibitory and excitatory innervation, and innervation from contralateral lumbar dI2 cells (Figure 7D and E). Thus, lumbar dI2 neurons can provide the cerebellum and the contralateral pre-MNs with proprioceptive information, copies of motor commands delivered from the ipsilateral pre-MNs, and integrated information from contralateral dI2 neurons (Figure 7D and E).

Our wiring-decoding studies are based on the availability of enhancer elements that direct expression in specific spinal INs. The lack of enhancer elements for known pre-MNs, such as V2, precluded their analysis. Future experiments using identified regulatory elements that direct expression in two other pre-MN populations, the dI3 and V0 neurons (Avraham et al., 2010b; Gard et al., 2017), will reveal the extent of premotor information relayed by dI2 neurons.

### dI2 subpopulations

Our study reveals two anatomically distinct subpopulations of dI2 neurons: precerebellar projection dI2 cells, which also innervate spinal targets along the entire extent of the spinal cord, and propriospinal dI2 cells, which innervate targets within the lumbar level. The laminar and medial/lateral positions of the two dI2 populations are similar, and we did not find subtype-specific expression of the known dI2 TFs. Contrary to our findings, a recent study (Osseward et al., 2021) has shown that tract and propriospinal INs in the mouse spinal cord differ in the localization of their somata along the mediolateral axis and their transcription of TFs. Tract neurons reside in the lateral spinal cord and express group-N TFs, while propriospinal neurons settle at the medial spinal cord and express group-Z TFs. Several reasons may explain the discrepancy between our results and those of Osseward et al. The analysis in mice (Osseward et al., 2021) was applied to numerous cardinal populations of neurons but excluded dI2 neurons. Hence, dI2 may represent an exception. In addition, our study revealed that precerebellar dI2 neurons share two wiring patterns: tract and propriospinal neurons. Applying single-cell RNA sequencing to dI2 neurons will reveal whether short- and long-range targeting in dI2 neurons are characterized by distinct transcriptomes or by a shared N + Z transcriptome.

### Physiological role of dI2 neurons

The unclear genetic origin of physiologically equivalent lumbar VSCT neurons has prevented a better understanding of their role in hindlimb locomotion. Our wiring and neuronal-silencing studies implicated dI2 as a significant contributor to the regularity and stability of locomotion in P8 hatchlings. The kinematic analysis of TeTX-treated hatchlings revealed imbalanced locomotion with occasional collapses, increased stride variability, a wide-base gait, and variable limb movements during stepping.

The mechanisms accounting for impaired stepping following dI2 neuron silencing are still unknown. One of the possible mechanisms is that silencing dI2 neurons perturbs the delivery of peripheral and intrinsic feedback to the cerebellum, leading to unreliable updating of the motor output produced by the locomotor networks, thereby impairing bipedal stepping. Another possible mechanism is based on the similarity of the gait instabilities of TeTX-treated hatchlings to ataxic motor disorders. Mammalian VSCT neurons receive descending input from reticulospinal, rubrospinal, and vestibulospinal pathways (Bras et al., 1988; Jankowska and Hammar, 2013). Neurons from the lateral vestibular nucleus have been reported to innervate extensor motoneurons at the lumbar level, as well as INs residing at medial lamina VII (Murray et al., 2018), the location where dI2 neurons were found to reside in our study. Thus, the vestibulospinal tract may convey input directly to the ipsilateral motor neurons and indirectly to contralateral motor neurons through dI2 neurons that innervate contralateral pre-MNs.

The local spinal connections between dI2 neurons and the contralateral pre-MNs and contralateral dI2 neurons may serve an important component of coordinated limb movements. dI2 synapses were found on both ipsilaterally and contralaterally projecting pre-MNs, both within their segmental level and at the brachial level, which regulates the movement of the wings. Thus, dI2 neurons may affect the motor output of the contralateral and ipsilateral sides of the cord by contacting commissural pre-MNs. Specific targeting of dI2 subpopulations – the precerebellar versus the propriospinal dI2 cells – is necessary to determine the relative contribution of dI2 subpopulations to the impaired stepping phenotype. However, there is no available genetic technique for differentially targeting the two subpopulations. In addition, the fact that the precerebellar dI2 neurons also innervate the lumbar spinal cord precludes the use of retrogradely target-derived neuronal activity modifiers.

In summary, our mapping studies of dI2 neurons and their connectomes, followed by characterization of the effects of their silencing on bipedal stepping, offer new insights into the function of dI2 neurons in vertebrates. We suggest that lumbar dI2 neurons not only relay sensory and intrinsic spinal network information to the cerebellum but also act as active mediators of motor functions at the lumbar segments and at the wing-controlling brachial segments of the spinal cord. Further circuit-deciphering studies of the constituents of subpopulations of dI2 cells, their targets, and their descending inputs are required to extend our understanding of the function of dI2 subpopulations in motor control.

## Materials and methods

**Key resources table:** 

Reagent type (species) or resource	Designation	Source or reference	Identifiers	Additional information
Strain, strain background (Chicken)	*Gallus gallus*	Gil-Guy Farm, Israel	NCBI Taxon: 9031	
Strain, strain background (*Pseudorabies virus*)	PRV152	Enquist and Card, 2003	NCBI Taxon: 10345	
Strain, strain background (*Pseudorabies virus*)	PRV614	Enquist and Card, 2003	NCBI Taxon: 10345	
Antibody	Rabbit anti-GFP(polyclonal)	Molecular Probes, Eugene, Oregon, USA	A-11122RRID:AB_221569	Dilution(1:1000)
Antibody	Mouse anti-GFP(monoclonal)	Abcam	Ab1218AB_298911	Dilution(1:100)
Antibody	Goat anti-GFP(polyclonal)	Abcam	Ab6673RRID:AB_305643	Dilution(1:300)
Antibody	Rabbit anti-RFP(polyclonal)	Acris	AP09229PU-NRRID:AB_2035909	Dilution(1:1000)
Antibody	Goat anti-ChAT(polyclonal)	Millipore, USA	AB144PRRID:AB_2079751	Dilution(1:300)
Antibody	Mouse anti-synaptotagmin(monoclonal)	Hybridoma Bank, University of Iowa, Iowa City, USA	ASV30RRID:AB_2295002	Dilution(1:100)
Antibody	Mouse anti-lhx1/5(monoclonal)	Hybridoma Bank, University of Iowa, Iowa City, USA	4F2RRID: AB_531784	Dilution(1:100)
Antibody	Mouse anti-FoxP4(monoclonal)	Hybridoma Bank, University of Iowa, Iowa City, USA	PCRP-FOXP4-1G7RRID:AB_2618641	Dilution(1:50)
Antibody	Rabbit anti-Pax2(polyclonal)	Abcam	ab79389RRID:AB_1603338	Dilution(1:50)
Antibody	Chicken anti- lacZ(polyclonal)	Abcam	ab79389RRID:AB_307210	Dilution(1:300)
Antibody	Rabbit anti-calbindin(polyclonal)	Swant	D-28kRRID:AB_2314070	Dilution(1:200)
Antibody	Goat anti-FoxP2(polyclonal)	Abcam	ab1307RRID:AB_1268914	Dilution(1:1000)
Antibody	Rabbit anti-5-HT(polyclonal)	Abcam	ab140495	Dilution(1:100)
Recombinant DNA reagent	EdI1::Cre	Avraham et al., 2009	N/A	
Recombinant DNA reagent	Ngn1::Cre	Avraham et al., 2009	N/A	
Recombinant DNA reagent	Ngn1::FLPo	Hadas et al., 2014	N/A	
Recombinant DNA reagent	Foxd3::FLPo	Hadas et al., 2014	N/A	
Recombinant DNA reagent	Foxd3::Cre	Avraham et al., 2009	N/A	
Recombinant DNA reagent	Isl1::Cre	Avraham et al., 2010a	N/A	
Recombinant DNA reagent	CAG-LSL-GFP	Hadas et al., 2014	N/A	
Recombinant DNA reagent	CAG-LSL-SV2-GFP	Hadas et al., 2014	N/A	
Recombinant DNA reagent	CAG-FSF-LSL-GFP	Hadas et al., 2014	N/A	
Recombinant DNA reagent	CAG-FSF-LSL-SV2-GFP	This paper	N/A	Figure 1—figure supplement 1;can be obtained from the Klar lab
Recombinant DNA reagent	CAG-FSF-LSL-cherry	This paper	N/A	Figure 1—figure supplement 1;can be obtained from the Klar lab
Recombinant DNA reagent	CAG-FSF-LSL-SV2-cherry	This paper	N/A	Figure 1—figure supplement 1;can be obtained from the Klar lab
Recombinant DNA reagent	CAG-FSF-LSL-AP	This paper	N/A	Figure 1—figure supplement 1;can be obtained from the Klar lab
Recombinant DNA reagent	CAG-LSL-TeXT	This paper	N/A	Figure 1—figure supplement 1;can be obtained from the Klar lab
Recombinant DNA reagent	CAG-LSL-F_SV2-cherry_F-GFP	This paper	N/A	Figure 1—figure supplement 1;can be obtained from the Klar lab
Recombinant DNA reagent	pGEMTEZ-TeTxLC	Addgene	#32640	
Sequence-based reagent	Foxd3-F	This paper	PCR primers	TCATCACCATGGCCATCCTG
Sequence-based reagent	Foxd3-R	This paper	PCR primers	GCTGGGCTCGGATTTCACGAT
Sequence-based reagent	vGlut2-F	This paper	PCR primers	GGAAGATGGGAAGCCCATGG
Sequence-based reagent	vGlut2-R	This paper	PCR primers	GAAGTCGGCAATTTGTCCCC
Sequence-based reagent	VIAAT-F	This paper	PCR primers	CTGAACGTCACCAACGCCATCC
Sequence-based reagent	VIAAT-R	This paper	PCR primers	GGGTAGGAGAGCAAGGCTTTG
Commercial assay or kit	NucleoBond Xtra Midi	Macherey-Nagel	Cat # 740410.50	
Chemical compound, drug	CTB conjugated to Alexa Fluor 647	Thermo Fisher	C34778	0.3 M
Software, algorithm	JMP	JMP	https://www.jmp.com/en_gb/home.html	
Software, algorithm	Adobe Photoshop	Adobe	https://www.adobe.com/il_en/	
Software, algorithm	ImageJ	ImageJ	https://imagej.nih.gov/ij/	
Software, algorithm	IMARIS	Oxford Instruments	https://imaris.oxinst.com/	
Software, algorithm	MacVector	MacVector	https://macvector.com/index.html	
Other(electroporator)	BTX Electroporator	BTX Harvard Apparatus	Cat#45-0662	
Other(confocal microscope)	FV1000; Olympus	Olympus	https://www.olympus-global.com/	
Other(microscope)	Eclipse Ni	Nikon	https://www.nikon.com/	
Other(light-sheet microscope)	LaVision Ultramicroscope II light-sheet microscope	LaVision BioTec	https://www.lavisionbiotec.com/	

### Animals

Fertilized white leghorn chicken eggs (Gil-Guy Farm, Israel) were incubated under standard conditions at 38°C. All experiments involving animals followed the designated policies of the Experiments in Animals Ethics Committee and were performed with its approval.

### 3D reconstruction and density plot analysis

The programs for both 3D reconstruction and the density plot analysis were written in MATLAB. The density plots were generated based on cross-sectional images converted to a standard form. The background was subtracted, and the cells were filtered automatically based on their soma size or using a manual approach. Subsequently, two-dimensional kernel density estimation was obtained using the MATLAB function ‘*kde2d*.’ Finally, unless indicated otherwise, a contour plot was drawn for density values between 20% and 80% of the estimated density range, with six contour lines.

### In ovo electroporation

A DNA solution of 5 mg/mL was injected into the lumen of the neural tube at HH stage 17–18 (E2.75–E3). Electroporation was performed using 3 × 50 ms pulses at 25–30 V, applied across the embryo using a 0.5 mm tungsten wire and a BTX electroporator (ECM 830). Following electroporation, 150–300 μL of antibiotic solution containing 100 unit/mL penicillin in Hanks’ balanced salt solution (Biological Industry, Beit-Haemek) was added on top of the embryos. Embryos were incubated for 3–19 days prior to further treatment or analysis.

### Immunohistochemistry and in situ hybridization

Embryos were fixed overnight at 4°C in 4% paraformaldehyde/0.1 M phosphate buffer, washed twice with phosphate buffered saline (PBS), incubated in 30% sucrose/PBS for 24 hr, and embedded in optimal cutting temperature (OCT) compound (Scigen, Grandad, USA). Sections with a thickness of 20 μm were cut on a cryostat. These sections were collected on Superfrost Plus slides and kept at −20°C. For 100 μm sections, spinal cords were isolated from the fixed embryos and subsequently embedded in warm 5% agar (in PBS), and 100 μm sections (E12–E17) were cut with a vibratome. Sections were collected in wells (free-floating technique) and processed for immunolabeling.

The following primary antibodies were used: rabbit polyclonal GFP antibody 1:1000 (Molecular Probes, Eugene, OR, USA), mouse anti-GFP 1:100, goat anti-GFP 1:300 (Abcam), rabbit anti-RFP 1:1000 (Acris), goat anti-ChAT antibody 1:300 (Cemicon, Temecula, CA, USA), mouse anti-synaptotagmin antibody 1:100 (ASV30), mouse anti-Lhx1/5 1:100 (4F2), mouse anti-FoxP4 1:50 (hybridoma bank, University of Iowa, Iowa City, USA), mouse anti-Brn3a 1:50 (Mercury), rabbit anti-Pax2 antibody 1:50 (Abcam), chicken anti-lacZ antibody 1:300 (Abcam), rabbit anti-Calbindin 1:200 (Swant), rabbit anti-VGLUT2 antibody (Synaptic Systems, Göttingen, Germany), goat anti-FoxP2:1000 (Abcam), anti-FoxP1:100 (ABR Synaptic), and rabbit anti-5-HT (Abcam). The following secondary antibodies were used: Alexa Fluor 488/647-conjugated AffiniPure donkey anti-mouse, anti-rabbit, and anti-goat (Jackson) and Rhodamine Red-X-conjugated donkey anti-mouse and anti-rabbit (Jackson). Images were taken under a microscope (Eclipse Ni; Nikon) with a digital camera (Zyla sCMOS; Andor) or captured using the integrated camera of a confocal microscope (FV1000; Olympus).

In situ hybridization was performed as previously described (Avraham et al., 2010a). The following probes were employed: Foxd3, vGlut2, and VIAAT probes were amplified from the cDNA of E6 chick embryos using the following primers. Foxd3: forward TCATCACCATGGCCATCCTG, reverse GCTGGGCTCGGATTTCACGAT. vGlut2: forward GGAAGATGGGAAGCCCATGG, and reverse GAAGTCGGCAATTTGTCCCC. VIAAT: forward CTGAACGTCACCAACGCCATCC, reverse GGGTAGGAGAGCAAGGCTTTG. The T7 RNA polymerase cis-binding sequence was added to the reverse primers.

### Laminar division

The standard forms of the spinal cord (for the crural, sciatic, and brachial plexus levels) were computationally divided into polygons for the different laminae (Martin, 1979). The number of neurons or synapses inside each lamina border was scored using their coordinates.

Light-sheet microscopy dI2::mCherry was electroporated into the embryos at HH stage 17–18. Embryos were removed at E13, and the spinal cord and cerebellum were isolated. The tissue was cleared using the iDISCO technique as described (Renier et al., 2014). The mCherry-expressing neurons were stained by application of an anti-RFP antibody followed by Rhodamine Red-X-conjugated donkey secondary antibody. Each staining step included 3 days of incubation with the antibody and subsequent washing for 2 days. Then, the cleared tissue was divided into three segments: a lumbar spinal segment, a brachial spinal segment, and a segment including the brainstem and cerebellum. Each sample was placed in a quartz imaging chamber (LaVision BioTec) and scanned by a LaVision Ultramicroscope II light-sheet microscope operated by ImspectorPro software (LaVision BioTec). An Andor Neo sCMOS camera was used for 16-bit image acquisition. The imaging was performed at 2× magnification with a 0.5–1 µm step size and a green excitation filter (peak – 525 nm/width − 50 nm). Then, for 3D reconstruction and analysis of the samples, the resulting image z-stacks were converted to IMS format using Imaris File Converter (version 9.5). Because of the sample size, several z-stacks were required for full acquisition of each sample; they were stitched together into one z-stack by Imaris Stitcher. Then, the files were uploaded to Imaris (9.6 version) for advanced visualization and analysis. dI2 axons were tracked using the filament tracer feature in semiautomatic mode. After tracking, Imaris was used to generate videos and snapshots describing different features of the analyzed samples. Finally, text, arrows, and other symbols were added using Adobe AfterEffects software.

### Synaptic marker validation

Validation of SV2-GFP reporter specificity was performed by using Imaris software. High-resolution confocal images of spinal cord sections, with clear SV2-GFP reporter expression and synaptotagmin (syn) immunolabeling, were used to quantify the degree of overlap of GFP^+^ terminals and syn^+^-labeled boutons. Both signals were three-dimensionally reconstructed, and we used the automatic quantification abilities of Imaris, further validated by additional manual counting, to quantify the number of GFP^+^ presynaptic terminals containing at least one syn^+^ bouton. In addition, the volume of GFP^+^ presynaptic terminals was documented to explore a possible dependence between terminal volume and syn^+^ bouton containment.

### AP staining

The treated embryos were fixed with 4% paraformaldehyde–PBS for 24 hr at 4°C and washed twice with PBS for 30 min at 4°C. The fixed embryos were incubated at 65°C in PBS for 8–16 hr to inactivate endogenous AP activity. The treated embryos were washed with 100 mM Tris–Cl (pH 9.5) containing 100 mM NaCl and 50 mM MgCl_2_, and the residual placental alkaline phosphatase activity was visualized by incubating the embryos with NBT/BCIP (Roche) in the same buffer at 4°C for 24 hr. After extensively washing the embryos with PBS–5 mM EDTA, the spinal cord was imaged.

### PRV infection and CTB retrograde labeling

From the attenuated PRV Bartha strain, we used two isogenic recombinants that express enhanced GFP (PRV152) and monomeric red fluorescent protein (PRV614). The viruses were harvested from Vero cell cultures at titers of 4 × 10^8^, 7 × 10^8^, and 1 × 10^9^ plaque-forming units (PFU/mL). Viral stocks were stored at −80°C. Injections of 3 μL of PRV152 or PRV614 were made into the thigh, pectoralis, or distal wing musculature of E13 or E14 chick embryos using a Hamilton syringe (Hamilton; Reno, NV, USA) equipped with a 33-gauge needle. The embryos were incubated for 36–40 hr and sacrificed for analysis. For spinocerebellar projecting neuron labeling, we used a replication-defective HSV (TK^-^) that contains a lacZ reporter. The virus was injected into the cerebellum of E12–15 embryos in ovo, and the embryos were incubated for another 40–48 hr. Alternatively, CTB conjugated to Alexa Fluor 647 (Thermo Fisher) was injected into the cerebellum of E12–15 embryos together with the virus for visualization of both precerebellular neurons and the upstream neurons.

### Force test

Muscle strength was evaluated using the measurement of the slope at which the chicks fell from a mesh surface as it was gradually tilted up from the horizontal. This test was repeated for each chick at least three times, and the average falling angle was calculated.

### Analysis of left-right phase

Stride duration was measured as the time from right toe-off/foot-off to the next right toe-off (as a complete stride cycle for the right leg), and the ‘half-cycle’ duration was measured as the time from right toe-off to the time of left toe-off. The following formula was used to calculate the phase: ((LeftToeOff_1 - RightToeOff_1)/(RightToeOff_2 – RightToeOff_1))*360.

### Behavioral tests and analysis

The embryos were bilaterally electroporated and then allowed to develop and hatch in a properly humidified and heated incubator. Afterwards, within 32 hr after hatching, the hatchling chicks were imprinted on the trainer. The P8 chicks were filmed in slow motion (240 fps) while freely walking (side and top views). The following parameters were scored: (1) weight; (2) foot grip strength; and (3) kinematic parameters during overground locomotion: (a) swing velocity, (b) swing and stance duration, (c) phase of footfalls, (d) heights of the knee and TMP joints, (e) angles of the TMP and ankle joints, (f) stride width (distance between feet during the double stance phase), and (g) landing angle.

Using semiautomated MATLAB-based tracking software (Hedrick, 2008), several points of interest were encoded. The leg joints as well as the eye and the tail were tracked. The position of these reference points was used for computational analysis using in-house MATLAB code for calculating different basic locomotion parameters (e.g., stick diagrams, velocity, joint trajectory, angles, range, and elevation), step patterns, and degrees of similarity. The landing angle was calculated as the angle between the imaginary line connecting the knee and the TMP joints and the ground, at the end of the swing phase. Dunnett’s test (Dunnett, 1955) was used to perform multiple comparisons of group means following one-way ANOVA. Circular statistics were used for analyses of angular data utilizing Oriana (KCS, version 4).

## Data Availability

All data generated or analysed during this study are included in the manuscript and the supporting files.

## Additional files

Supplementary file 1. Weight, force, and number of electroporated cells. See statistical tests in Supplementary Statistical analysis tables.

Transparent reporting form

## References

[bib1] Alaynick WA, Jessell TM, Pfaff SL (2011). Snapshot: Spinal cord development. Cell.

[bib2] Andersson LS, Larhammar M, Memic F, Wootz H, Schwochow D, Rubin CJ, Patra K, Arnason T, Wellbring L, Hjälm G, Imsland F, Petersen JL, McCue ME, Mickelson JR, Cothran G, Ahituv N, Roepstorff L, Mikko S, Vallstedt A, Lindgren G, Andersson L, Kullander K (2012). Mutations in DMRT3 affect locomotion in horses and spinal circuit function in mice. Nature.

[bib3] Avraham O, Hadas Y, Vald L, Zisman S, Schejter A, Visel A, Klar A (2009). Transcriptional control of axonal guidance and sorting in dorsal interneurons by the LIM-HD proteins lhx9 and lhx1. Neural Development.

[bib4] Avraham O, Hadas Y, Vald L, Hong S, Song MR, Klar A (2010a). Motor and dorsal root ganglion axons serve as choice points for the ipsilateral turning of di3 axons. The Journal of Neuroscience.

[bib5] Avraham O, Zisman S, Hadas Y, Vald L, Klar A (2010b). Deciphering axonal pathways of genetically defined groups of neurons in the chick neural tube utilizing in ovo electroporation. Journal of Visualized Experiments.

[bib6] Belle M, Godefroy D, Dominici C, Heitz-Marchaland C, Zelina P, Hellal F, Bradke F, Chédotal A (2014). A simple method for 3d analysis of immunolabeled axonal tracts in a transparent nervous system. Cell Reports.

[bib7] Bermingham NA, Hassan BA, Wang VY, Fernandez M, Banfi S, Bellen HJ, Fritzsch B, Zoghbi HY (2001). Proprioceptor pathway development is dependent on math1. Neuron.

[bib8] Bikoff JB, Gabitto MI, Rivard AF, Drobac E, Machado TA, Miri A, Brenner-Morton S, Famojure E, Diaz C, Alvarez FJ, Mentis GZ, Jessell TM (2016). Spinal inhibitory interneuron diversity delineates variant motor microcircuits. Cell.

[bib9] Bras H, Cavallari P, Jankowska E (1988). Demonstration of initial axon collaterals of cells of origin of the ventral spinocerebellar tract in the cat. The Journal of Comparative Neurology.

[bib10] Bui TV, Akay T, Loubani O, Hnasko TS, Jessell TM, Brownstone RM (2013). Circuits for grasping: Spinal di3 interneurons mediate cutaneous control of motor behavior. Neuron.

[bib11] Cheng L, Arata A, Mizuguchi R, Qian Y, Karunaratne A, Gray PA, Arata S, Shirasawa S, Bouchard M, Luo P, Chen CL, Busslinger M, Goulding M, Onimaru H, Ma Q (2004). Tlx3 and tlx1 are post-mitotic selector genes determining glutamatergic over gabaergic cell fates. Nature Neuroscience.

[bib12] Clark AJ, Higham TE (2011). Slipping, sliding and stability: Locomotor strategies for overcoming low-friction surfaces. The Journal of Experimental Biology.

[bib13] Daley MA, Biewener AA (2006). Running over rough terrain reveals limb control for intrinsic stability. PNAS.

[bib14] Delile J, Rayon T, Melchionda M, Edwards A, Briscoe J, Sagner A (2019). Single cell transcriptomics reveals spatial and temporal dynamics of gene expression in the developing mouse spinal cord. Development.

[bib15] Dunnett CW (1955). A multiple comparison procedure for comparing several treatments with a control. Journal of the American Statistical Association.

[bib16] Enquist LW, Card JP (2003). Recent advances in the use of neurotropic viruses for circuit analysis. Current Opinion in Neurobiology.

[bib17] Francius C, Harris A, Rucchin V, Hendricks TJ, Stam FJ, Barber M, Kurek D, Grosveld FG, Pierani A, Goulding M, Clotman F (2013). Identification of multiple subsets of ventral interneurons and differential distribution along the rostrocaudal axis of the developing spinal cord. PLOS ONE.

[bib18] Furue M, Uchida S, Shinozaki A, Imagawa T, Hosaka YZ, Uehara M (2010). Spinocerebellar projections from the cervical and lumbosacral enlargements in the chicken spinal cord. Brain, Behavior and Evolution.

[bib19] Furue M, Uchida S, Shinozaki A, Imagawa T, Hosaka YZ, Uehara M (2011). Trajectories in the spinal cord and the mediolateral spread in the cerebellar cortex of spinocerebellar fibers from the unilateral lumbosacral enlargement in the chicken. Brain, Behavior and Evolution.

[bib20] Gard C, Gonzalez Curto G, Frarma YEM, Chollet E, Duval N, Auzié V, Auradé F, Vigier L, Relaix F, Pierani A, Causeret F, Ribes V (2017). Pax3- and pax7-mediated dbx1 regulation orchestrates the patterning of intermediate spinal interneurons. Developmental Biology.

[bib21] Gosgnach S, Lanuza GM, Butt SJB, Saueressig H, Zhang Y, Velasquez T, Riethmacher D, Callaway EM, Kiehn O, Goulding M (2006). V1 spinal neurons regulate the speed of vertebrate locomotor outputs. Nature.

[bib22] Griener A, Zhang W, Kao H, Wagner C, Gosgnach S (2015). Probing diversity within subpopulations of locomotor-related v0 interneurons. Developmental Neurobiology.

[bib23] Hadas Y, Etlin A, Falk H, Avraham O, Kobiler O, Panet A, Lev-Tov A, Klar A (2014). A “tool box” for deciphering neuronal circuits in the developing chick spinal cord. Nucleic Acids Research.

[bib24] Hammar I, Maxwell DJ (2002). Serotoninergic and noradrenergic axons make contacts with neurons of the ventral spinocerebellar tract in the cat. The Journal of Comparative Neurology.

[bib25] Hammar I, Bannatyne BA, Maxwell DJ, Edgley SA, Jankowska E (2004). The actions of monoamines and distribution of noradrenergic and serotoninergic contacts on different subpopulations of commissural interneurons in the cat spinal cord. The European Journal of Neuroscience.

[bib26] Hantman AW, Jessell TM (2010). Clarke’s column neurons as the focus of a corticospinal corollary circuit. Nature Neuroscience.

[bib27] Hedrick TL (2008). Software techniques for two- and three-dimensional kinematic measurements of biological and biomimetic systems. Bioinspiration & Biomimetics.

[bib28] Jankowska E, Hammar I (2013). Interactions between spinal interneurons and ventral spinocerebellar tract neurons. The Journal of Physiology.

[bib29] Jessell TM (2000). Neuronal specification in the spinal cord: Inductive signals and transcriptional codes. Nature Reviews. Genetics.

[bib30] Jiang J, Azim E, Ekerot CF, Alstermark B (2015). Direct and indirect spino-cerebellar pathways: Shared ideas but different functions in motor control. Frontiers in Computational Neuroscience.

[bib31] Lai HC, Seal RP, Johnson JE (2016). Making sense out of spinal cord somatosensory development. Development.

[bib32] Martin AH (1979). A cytoarchitectonic scheme for the spinal cord of the domestic fowl, Gallus gallus domesticus: Lumbar region. Acta Morphologica Neerlando-Scandinavica.

[bib33] Morikawa Y, Hisaoka T, Senba E (2009). Characterization of foxp2-expressing cells in the developing spinal cord. Neuroscience.

[bib34] Murray AJ, Croce K, Belton T, Akay T, Jessell TM (2018). Balance control mediated by vestibular circuits directing limb extension or antagonist muscle co-activation. Cell Reports.

[bib35] Osseward PJ, Pfaff SL (2019). Cell type and circuit modules in the spinal cord. Current Opinion in Neurobiology.

[bib36] Osseward PJ, Amin ND, Moore JD, Temple BA, Barriga BK, Bachmann LC, Beltran F, Gullo M, Clark RC, Driscoll SP, Pfaff SL, Hayashi M (2021). Conserved genetic signatures parcellate cardinal spinal neuron classes into local and projection subsets. Science.

[bib37] Renier N, Wu Z, Simon DJ, Yang J, Ariel P, Tessier-Lavigne M (2014). IDISCO: A simple, rapid method to immunolabel large tissue samples for volume imaging. Cell.

[bib38] Ruder L, Takeoka A, Arber S (2016). Long-distance descending spinal neurons ensure quadrupedal locomotor stability. Neuron.

[bib39] Sakai N, Insolera R, Sillitoe RV, Shi SH, Kaprielian Z (2012). Axon sorting within the spinal cord marginal zone via robo-mediated inhibition of n-cadherin controls spinocerebellar tract formation. The Journal of Neuroscience.

[bib40] Spanne A, Jörntell H (2013). Processing of multi-dimensional sensorimotor information in the spinal and cerebellar neuronal circuitry: A new hypothesis. PLOS Computational Biology.

[bib41] Stecina K, Fedirchuk B, Hultborn H (2013). Information to cerebellum on spinal motor networks mediated by the dorsal spinocerebellar tract. The Journal of Physiology.

[bib42] Sweeney LB, Bikoff JB, Gabitto MI, Brenner-Morton S, Baek M, Yang JH, Tabak EG, Dasen JS, Kintner CR, Jessell TM (2018). Origin and segmental diversity of spinal inhibitory interneurons. Neuron.

[bib43] Uehara M, Akita M, Furue M, Shinozaki A, Hosaka YZ (2012). Laterality of spinocerebellar neurons in the chicken spinal cord. The Journal of Veterinary Medical Science.

[bib44] Uemura O, Okada Y, Ando H, Guedj M, Higashijima SI, Shimazaki T, Chino N, Okano H, Okamoto H (2005). Comparative functional genomics revealed conservation and diversification of three enhancers of the isl1 gene for motor and sensory neuron-specific expression. Developmental Biology.

[bib45] Valenzuela JI, Hasan SJ, Steeves JD (1990). Stimulation of the brainstem reticular formation evokes locomotor activity in embryonic chicken (in ovo. Brain Research. Developmental Brain Research.

[bib46] Yamamoto M, Wada N, Kitabatake Y, Watanabe D, Anzai M, Yokoyama M, Teranishi Y, Nakanishi S (2003). Reversible suppression of glutamatergic neurotransmission of cerebellar granule cells in vivo by genetically manipulated expression of tetanus neurotoxin light chain. The Journal of Neuroscience.

[bib47] Yuengert R, Hori K, Kibodeaux EE, McClellan JX, Morales JE, Huang TWP, Neul JL, Lai HC (2015). Origin of a Non-Clarke’s Column Division of the Dorsal Spinocerebellar Tract and the Role of Caudal Proprioceptive Neurons in Motor Function. Cell Reports.

