# Supplementary material for: Spinal lumbar dI2 interneurons contribute to stability of bipedal stepping
Source: eLife. 2021 Aug 16;10:e62001. doi: 10.7554/eLife.62001 (PMC8448531; doi:10.7554/eLife.62001)
Supplement: Figure 6—source data 3. — Statistical analysis for the data presented in Figure 6. [file elife-62001-fig6-data3.docx]

**Figure 6-source data 3**

Statistical analysis for the data presented in Figure 6

Fig. 6A: Body Collapses.

**Pooled data**

| Z test results |  |  |  |
| --- | --- | --- | --- |
|  |  | Z score | p Value |
| Control | TeTX | -7.0147 | <0.00001 |

**Pairwise Comparisons**

| Z test results |  |  |  |
| --- | --- | --- | --- |
|  |  | Z score | p Value |
| Control1 | Control2 | 0.6395 | 0.52218 |
| Control1 | Control3 | 1.6064 | 0.1704 |
| Control1 | Control4 | 1.0451 | 0.29372 |
| Control2 | Control3 | 0 | 1 |
| Control2 | Control4 | 0 | 1 |
| Control3 | Control4 | 0 | 1 |
| Control1 | TeTX1 | -0.6932 | 0.4902 |
| Control1 | TeTX2 | -2.8791 | 0.00398 |
| Control1 | TeTX3 | -2.7099 | 0.00672 |
| Control1 | TeTX4 | -3.2439 | 0.0012 |
| Control1 | TeTX5 | -3.7566 | 0.00016 |
| Control2 | TeTX1 | -0.9321 | 0.35238 |
| Control2 | TeTX2 | -2.1564 | 0.03078 |
| Control2 | TeTX3 | -2.0468 | 0.04036 |
| Control2 | TeTX4 | -2.39 | 0.01684 |
| Control2 | TeTX5 | -2.694 | 0.00714 |
| Control3 | TeTX1 | -2.3323 | 0.0198 |
| Control3 | TeTX2 | -5.1786 | <0.00001 |
| Control3 | TeTX3 | -4.9411 | <0.00001 |
| Control3 | TeTX4 | -5.6775 | <0.00001 |
| Control3 | TeTX5 | -6.3364 | <0.00001 |
| Control4 | TeTX1 | -1.5212 | 0.12852 |
| Control4 | TeTX2 | -3.4432 | 0.00058 |
| Control4 | TeTX3 | -3.2787 | 0.00104 |
| Control4 | TeTX4 | -3.7928 | 0.00016 |
| Control4 | TeTX5 | -4.266 | <0.00001 |

Fig. 6B: Statistical analysis

Analysis of Range (Knee Height)

**Quantiles**

| **Level** | **Minimum** | **10%** | **25%** | **Median** | **75%** | **90%** | **Maximum** |
| --- | --- | --- | --- | --- | --- | --- | --- |
| Control | 1.356529 | 1.605726 | 1.732048 | 1.883241 | 2.196335 | 2.512926 | 3.01324 |
| TeXT | 1.739098 | 2.260857 | 2.532573 | 3.146695 | 3.385301 | 4.151452 | 5.048257 |

**Means and Std Deviations**

| **Level** | **Number** | **Mean** | **Std Dev** | **Std Err Mean** | **Lower 95%** | **Upper 95%** |
| --- | --- | --- | --- | --- | --- | --- |
| Control | 504 | 1.9810563 | 0.33479 | 0.0149127 | 1.9517574 | 2.0103553 |
| TeXT | 630 | 3.1088092 | 0.7461462 | 0.0297272 | 3.0504327 | 3.1671857 |

**Tests that the Variances are Equal**

| **Level** | **Count** | **Std Dev** | **MeanAbsDif to Mean** | **MeanAbsDif to Median** |
| --- | --- | --- | --- | --- |
| Control | 504 | 0.3347900 | 0.2772036 | 0.2696084 |
| TeXT | 630 | 0.7461462 | 0.5714468 | 0.5700685 |

| **Test** | **F Ratio** | **DFNum** | **DFDen** | **p-Value** |
| --- | --- | --- | --- | --- |
| O'Brien[.5] | 132.3435 | 1 | 1132 | <.0001* |
| Brown-Forsythe | 167.4121 | 1 | 1132 | <.0001* |
| Levene | 169.2823 | 1 | 1132 | <.0001* |
| Bartlett | 309.7565 | 1 | . | <.0001* |
| F Test 2-sided | 4.9671 | 629 | 503 | <.0001* |

**Welch's Test**

Welch Anova testing Means Equal, allowing Std Devs Not Equal

| **F Ratio** | **DFNum** | **DFDen** | **Prob > F** |
| --- | --- | --- | --- |
| 1149.8353 | 1 | 913.1 | <.0001* |

| **t Test** |
| --- |
| 33.9092 |

Fig. 6C: Statistical analysis

Analysis of Range (TMP Angle)

| WATSON-WILLIAMS F-TESTS |  |  |  |  |  |
| --- | --- | --- | --- | --- | --- |
| Variables (& observations) | F | p | df | df2 | Est. Mean |
| Controls & TeTX (504 & 630) | 430.895 | < 1E-12 | 1 | 1132 | 62.188 |
