# Supplementary material for: Spinal lumbar dI2 interneurons contribute to stability of bipedal stepping
Source: eLife. 2021 Aug 16;10:e62001. doi: 10.7554/eLife.62001 (PMC8448531; doi:10.7554/eLife.62001)
Supplement: Figure 7—figure supplement 1—source data 1. — Statistical analysis for the data presented in Figure 7—figure supplement 1. [file elife-62001-fig7-figsupp1-data1.docx]

**Figure 7-supp 1-source data 1**

Statistical analyses of the data presented in Figure 7-figure supplement 1

Figure 7-figure supplement 1A – Ranges of knee height

**Quantiles**

| **Level** | **Minimum** | **10%** | **25%** | **Median** | **75%** | **90%** | **Maximum** |
| --- | --- | --- | --- | --- | --- | --- | --- |
| Control_GFP1 | 1.583211 | 1.642068 | 1.732058 | 1.853918 | 2.036332 | 2.302025 | 2.338401 |
| Control_GFP2 | 1.356529 | 1.505224 | 1.553307 | 1.852974 | 2.061026 | 2.171198 | 2.220186 |
| Control_NoElec1 | 2.151395 | 2.170454 | 2.196808 | 2.381171 | 2.644546 | 2.742689 | 3.01324 |
| Control_NoElec2 | 1.574461 | 1.606336 | 1.661235 | 1.781316 | 1.840663 | 1.870193 | 1.914784 |
| TeTX1 | 2.221573 | 2.267482 | 2.648965 | 3.163871 | 3.358293 | 3.798085 | 3.948563 |
| TeTX2 | 3.180134 | 3.230696 | 3.278528 | 3.375239 | 3.61459 | 4.067007 | 4.314338 |
| TeTX3 | 2.190001 | 2.274949 | 2.343899 | 2.461626 | 2.703694 | 3.196665 | 3.322023 |
| TeTX4 | 1.739098 | 1.764574 | 1.977728 | 2.614597 | 2.978234 | 3.317682 | 3.538927 |
| TeTX5 | 2.771483 | 2.882091 | 3.133971 | 3.380367 | 4.820164 | 5.003136 | 5.048257 |

**Oneway Anova**

**Summary of Fit**

| Rsquare | 0.732832 |
| --- | --- |
| Adj Rsquare | 0.730932 |
| Root Mean Square Error | 0.425585 |
| Mean of Response | 2.607586 |
| Observations (or Sum Wgts) | 1134 |

**Analysis of Variance**

| **Source** | **DF** | **Sum of Squares** | **Mean Square** | **F Ratio** | **Prob > F** |
| --- | --- | --- | --- | --- | --- |
| Label | 8 | 558.91272 | 69.8641 | 385.7283 | <.0001* |
| Error | 1125 | 203.76287 | 0.1811 |  |  |
| C. Total | 1133 | 762.67560 |  |  |  |

**Means for Oneway Anova**

| **Level** | **Number** | **Mean** | **Std Error** | **Lower 95%** | **Upper 95%** |
| --- | --- | --- | --- | --- | --- |
| Control_GFP1 | 126 | 1.91458 | 0.03791 | 1.8402 | 1.9890 |
| Control_GFP2 | 126 | 1.83644 | 0.03791 | 1.7620 | 1.9108 |
| Control_NoElec1 | 126 | 2.41935 | 0.03791 | 2.3450 | 2.4937 |
| Control_NoElec2 | 126 | 1.75386 | 0.03791 | 1.6795 | 1.8282 |
| TeTX1 | 126 | 3.05139 | 0.03791 | 2.9770 | 3.1258 |
| TeTX2 | 126 | 3.50734 | 0.03791 | 3.4330 | 3.5817 |
| TeTX3 | 126 | 2.57487 | 0.03791 | 2.5005 | 2.6493 |
| TeTX4 | 126 | 2.54391 | 0.03791 | 2.4695 | 2.6183 |
| TeTX5 | 126 | 3.86654 | 0.03791 | 3.7921 | 3.9409 |

Figure 7-figure supplement 1B– Ranges of TMP joint

| BASIC STATISTICS |  |  |  |  |  |  |  |  |  |
| --- | --- | --- | --- | --- | --- | --- | --- | --- | --- |
| Variable | Control1 | Control2 | Control3 | Control4 | TeTX1 | TeTX2 | TeTX3 | TeTX4 | TeTX5 |
| Data Type | Angles | Angles | Angles | Angles | Angles | Angles | Angles | Angles | Angles |
| Number of Observations | 126 | 126 | 126 | 126 | 126 | 126 | 126 | 126 | 126 |
| Mean Vector (µ) | 56.804 | 41.427 | 54.864 | 44.118 | 82.279 | 71.793 | 64.171 | 72.488 | 72.866 |
| Circular Standard Deviation | 16.583 | 18.673 | 9.687 | 12.587 | 22.21 | 25.234 | 21.269 | 17.213 | 12.024 |

| WATSON-WILLIAMS F-TESTS |  |  |  |  |  |
| --- | --- | --- | --- | --- | --- |
| Variables (& observations) | F | p | df | df2 | Est. Mean |
| Multi-sample test using: |  |  |  |  |  |
| Control1 (126) |  |  |  |  |  |
| Control2 (126) |  |  |  |  |  |
| Control3 (126) |  |  |  |  |  |
| Control4 (126) |  |  |  |  |  |
| TeTX1 (126) |  |  |  |  |  |
| TeTX2 (126) |  |  |  |  |  |
| TeTX3 (126) |  |  |  |  |  |
| TeTX4 (126) |  |  |  |  |  |
| TeTX5 (126) | 76.168 | < 1E-12 | 8 | 1125 | 62.188 |
