# Supplementary material for: Spinal lumbar dI2 interneurons contribute to stability of bipedal stepping
Source: eLife. 2021 Aug 16;10:e62001. doi: 10.7554/eLife.62001 (PMC8448531; doi:10.7554/eLife.62001)
Supplement: Supplementary file 1. — See statistical tests in Supplementary Statistical analysis tables. [file elife-62001-supp1.docx]

Spinal lumbar dI2 interneurons contribute to stability of bipedal stepping

Supplementary Information

**Supplementary file 1**

Weight, force and number of electroporated cells. See statistical tests in Supplementary Statistical analysis tables.

.

| chick | # of dI2::TeTX cells | % of large diameter dI2::TeTX cells | Weight in gr. | Force test  angle of fall | |
| --- | --- | --- | --- | --- | --- |
|  |  |  |  | Mean ± circ*SD* | *N* |
| TeTX1 | 602 | 5.64 | 139 | 68.41 ± 2.8 | 3 |
| TeTX2 | 124 | 6.45 | 139 | 63.34 ± 1.85 | 3 |
| TeTX3 | 81 | 7.4 | 159 | 65.45 ± 3.35 | 5 |
| TeTX4 | 755 | 7.01 | 148 | 69.29 ± 3.48 | 5 |
| TeTX5 | 769 | 8.71 | 139 | 66.44 ± 3.75 | 3 |
| Control 3 |  |  | 158 | 64.21 ± 2 | 16 |
| Control 4 |  |  | 164 | 63.08 ± 1.89 | 12 |
| Control 5 |  |  | 144 | 66.93 ± 3.59 | 6 |
| Control 6 |  |  | 135 | 64.61 ± 2.57 | 6 |
| Control 7 |  |  | 122 | 66.11 ± 5.57 | 7 |
