# Supplementary material for: Spinal lumbar dI2 interneurons contribute to stability of bipedal stepping
Source: eLife. 2021 Aug 16;10:e62001. doi: 10.7554/eLife.62001 (PMC8448531; doi:10.7554/eLife.62001)
Supplement: Transparent reporting form [file elife-62001-transrepform1.docx]

***eLife’s* transparent reporting form**

We encourage authors to provide detailed information *within their submission* to facilitate the interpretation and replication of experiments. Authors can upload supporting documentation to indicate the use of appropriate reporting guidelines for health-related research (see [EQUATOR Network](http://www.equator-network.org/%20)), life science research (see the [BioSharing Information Resource](https://biosharing.org/)), or the [ARRIVE guidelines](http://www.plosbiology.org/article/info:doi/10.1371/journal.pbio.1000412) for reporting work involving animal research. Where applicable, authors should refer to any relevant reporting standards documents in this form.

If you have any questions, please consult our Journal Policies and/or contact us: [editorial@elifesciences.org](mailto:editorial@elifesciences.org).

**Sample-size estimation**

- You should state whether an appropriate sample size was computed when the study was being designed
- You should state the statistical method of sample size computation and any required assumptions
- If no explicit power analysis was used, you should describe how you decided what sample (replicate) size (number) to use

Please outline where this information can be found within the submission (e.g., sections or figure legends), or explain why this information doesn’t apply to your submission:

Fig. 1F and 1I: The number of embryos and the number of cell (N) are indicated in the figure legend. Page 7 and in page 36 lines 645-660.

Fig. 2E,G,H: The number of embryos and the number of cell (N) are indicated in the figure legend. Page 10 and in page 37 lines 674-681.

Fig. 3: A spinal cord of one E13 embryo.

Fig. 4: The number of embryos and the number of cell (N) is indicated are the figure legend. Page 13 and in pages 37-38 lines 683-705.

Fig. 5: The number of embryos and the number of cell (N) are indicated in the figure legend. Page 15 and in pages 38-39 lines 707-733.

Fig. 6D: The number of profiles of steps for each chick are indicated in table 3. Page 23.

Fig. 7A: The number of steps that were analyzed are indicated in table 3. The statistical analysis in presented in the figure legend (page 20 and page 40 lines 756-758) and in supplementary data page 8.

Fig. 7B. The number of steps that were analyzed are indicated in table 3. The statistical analysis in presented in the figure legend (page 20 and page 40 lines 759-761) and in supplementary data page 9.

Fig. 7C. The number of steps that were analyzed are indicated in table 3. The statistical analysis in presented in the figure legend (page 20 and page 41 lines 763-765) and in supplementary data page 10.

Table 1 and Supp Fig. 8A: Statistical analysis in supplementary data page 13.

Table 2: Statistical analysis in supplementary data page 12.

Table 3 and Supp Fig. 8B,C: Statistical analysis in supplementary data pages 14-15.

Supp Fig. S2E: The number of embryos and the number of cell (N) are indicated in the Supp figure legend,

Supp Fig. 3A,B: The number of embryos and the number of cell (N) are indicated in the Supp figure legend,

Supp Fig. 4B-D: The number of embryos and the number of cell (N) are indicated in the Supp figure legend,

Supp Fig. 5D: The number of embryos and the number of cell (N) are indicated in the Supp figure legend,

Supp Fig. 6C: The number of embryos and the number of cell (N) are indicated in the Supp figure legend, pages 3-4.

Supp Fig. 7C: The number of embryos and the number of cell (N) are indicated in the Supp figure legend,

Supp Fig. 8C,D,E,F,G,H: The number of embryos and the number of cell (N) are indicated in the Supp figure legend, page .

Supp Fig. 9: The number of embryos and the number of cell (N) are indicated in the Supp figure legend, page .

Supp Fig. 10: The number of embryos and the number of cell (N) are indicated in the Supp figure legend, page .

Supp Fig. 11B,C: The number of embryos and the number of cell (N) are indicated in the Supp figure legend, page .

**Replicates**

- You should report how often each experiment was performed
- You should include a definition of biological versus technical replication
- The data obtained should be provided and sufficient information should be provided to indicate the number of independent biological and/or technical replicates
- If you encountered any outliers, you should describe how these were handled
- Criteria for exclusion/inclusion of data should be clearly stated
- High-throughput sequence data should be uploaded before submission, with a private link for reviewers provided (these are available from both GEO and ArrayExpress)

Please outline where this information can be found within the submission (e.g., sections or figure legends), or explain why this information doesn’t apply to your submission:

- You should report how often each experiment was performed

All the relevant information is within the above section - **Sample-size estimation**

- You should include a definition of biological versus technical replication

All the repeats (number of embryos, number of chicks, number of steps, number of cells, etc.) are biological replications.

- The data obtained should be provided and sufficient information should be provided to indicate the number of independent biological and/or technical replicates

All the relevant information is within the above section - **Sample-size estimation**

- If you encountered any outliers, you should describe how these were handled

All the data was analyzed. No outliers were excluded.

- Criteria for exclusion/inclusion of data should be clearly stated

All the data was analyzed. No outliers were excluded.

- High-throughput sequence data should be uploaded before submission, with a private link for reviewers provided (these are available from both GEO and ArrayExpress)

Not relevant.

**Statistical reporting**

- Statistical analysis methods should be described and justified
- Raw data should be presented in figures whenever informative to do so (typically when N per group is less than 10)
- For each experiment, you should identify the statistical tests used, exact values of N, definitions of center, methods of multiple test correction, and dispersion and precision measures (e.g., mean, median, SD, SEM, confidence intervals; and, for the major substantive results, a measure of effect size (e.g., Pearson's r, Cohen's d)
- Report exact p-values wherever possible alongside the summary statistics and 95% confidence intervals. These should be reported for all key questions and not only when the p-value is less than 0.05.

Please outline where this information can be found within the submission (e.g., sections or figure legends), or explain why this information doesn’t apply to your submission:

All the stat data is presented in the figure legends and the supplementary stat. For details see above in the **Sample-size estimation** section**.**

(For large datasets, or papers with a very large number of statistical tests, you may upload a single table file with tests, Ns, etc., with reference to sections in the manuscript.)

**Group allocation**

- Indicate how samples were allocated into experimental groups (in the case of clinical studies, please specify allocation to treatment method); if randomization was used, please also state if restricted randomization was applied
- Indicate if masking was used during group allocation, data collection and/or data analysis

Please outline where this information can be found within the submission (e.g., sections or figure legends), or explain why this information doesn’t apply to your submission:

Not relevant.

**Additional data files (“source data”)**

- We encourage you to upload relevant additional data files, such as numerical data that are represented as a graph in a figure, or as a summary table
- Where provided, these should be in the most useful format, and they can be uploaded as “Source data” files linked to a main figure or table
- Include model definition files including the full list of parameters used
- Include code used for data analysis (e.g., R, MatLab)
- Avoid stating that data files are “available upon request”

All the numerical data that are represented as graphs in the figures are presented in the supplementary statistics section with the supplementary data document.

Codes used for data analysis - <https://github.com/baruchhaimson/dI2-INs-codes-Elife>

Please indicate the figures or tables for which source data files have been provided:
